# Cilostazol for Secondary Prevention of Stroke and Cognitive Decline: Systematic Review and Meta-Analysis

**DOI:** 10.1161/STROKEAHA.120.029454

**Published:** 2020-07-10

**Authors:** Caroline McHutchison, Gordon W. Blair, Jason P. Appleton, Francesca M. Chappell, Fergus Doubal, Philip M. Bath, Joanna M. Wardlaw

**Affiliations:** 1 Centre for Clinical Brain Science, University of Edinburgh, United Kingdom (C.M., G.W.B., F.M.C., F.D.).; 2 Stroke Trials Unit, Division of Clinical Neuroscience, University of Nottingham, United Kingdom (J.P.A., P.M.B.).; 3 Department of Neurology, University Hospitals Birmingham NHS Foundation Trust, Mindelsohn Way, Edgbaston, United Kingdom (J.P.A.).

**Keywords:** aspirin, cilostazol, clopidogrel, meta-analysis, stroke, lacunar, stroke

## Abstract

**Background and Purpose:**

Cilostazol, a phosphodiesterase 3’ inhibitor, is used in Asia-Pacific countries for stroke prevention, but rarely used elsewhere. In addition to weak antiplatelet effects, it stabilizes endothelium, aids myelin repair and astrocyte-neuron energy transfer in laboratory models, effects that may be beneficial in preventing small vessel disease progression.

**Methods:**

A systematic review and meta-analysis of unconfounded randomized controlled trials of cilostazol to prevent stroke, cognitive decline, or radiological small vessel disease lesion progression. Two reviewers searched for papers (January 1, 2019 to July 16, 2019) and extracted data. We calculated Peto odds ratios (ORs) and 95% CIs for recurrent ischemic, hemorrhagic stroke, death, adverse symptoms, with sensitivity analyses. The review is registered (CRD42018084742).

**Results:**

We included 20 randomized controlled trials (n=10 505), 18 in ischemic stroke (total n=10 449) and 2 in cognitive impairment (n=56); most were performed in Asia-Pacific countries. Cilostazol decreased recurrent ischemic stroke (17 trials, n=10 225, OR=0.68 [95% CI, 0.57–0.81]; *P*<0.0001), hemorrhagic stroke (16 trials, n=9736, OR=0.43 [95% CI, 0.29–0.64]; *P*=0.0001), deaths (OR=0.64 [95% CI, 0.49–0.83], *P*<0.0009), systemic bleeding (n=8387, OR=0.73 [95% CI, 0.54–0.99]; *P*=0.04), but increased headache and palpitations, compared with placebo, aspirin, or clopidogrel. Cilostazol reduced recurrent ischemic stroke more when given long (>6 months) versus short term without increasing hemorrhage, and in trials with larger proportions (>40%) of lacunar stroke. Data were insufficient to assess effects on cognition, imaging, functional outcomes, or tolerance.

**Conclusions:**

Cilostazol appears effective for long-term secondary stroke prevention without increasing hemorrhage risk. However, most trials related to Asia-Pacific patients and more trials in Western countries should assess its effects on cognitive decline, functional outcome, and tolerance, particularly in lacunar stroke and other presentations of small vessel disease.

## Abstract 2

Supplemental Digital Content is available in the text.

Cerebral small vessel disease (SVD) causes 25% of ischemic stroke, most intracerebral hemorrhages, most vascular cognitive impairment and up to 45% of dementias, and other important aging-related comorbidities.^1^ There is no specific treatment to prevent SVD progression. In a review of SVDs mechanisms and therapeutic agents with relevant modes of action,^2^ we identified several licenced drugs including cilostazol, a phosphodiesterase 3′ inhibitor. In addition to mild antiplatelet effects,^3^ cilostazol has several actions targeting processes involved in SVD pathophysiology: endothelial dysfunction, myelin repair, neuroprotection, and inflammation.^2^

Cilostazol is used for stroke prevention in Asia-Pacific countries, but in Western countries it is used mostly for symptomatic peripheral vascular disease. Previous systematic reviews suggested that cilostazol prevented recurrent stroke.^4,5^ However, further trials have been published since the last review, no review has assessed cilostazol’s effects in relevant subgroups and few assessed adverse effects (bleeding, headaches, palpitations, etc) that could limit cilostazol tolerance.

We performed a systematic review and meta-analysis to determine the effect of cilostazol on stroke recurrence, cognitive decline, radiological progression of SVD, intracerebral hemorrhage, death and adverse symptoms in patients with stroke or cognitive presentations of SVD.

## Methods

We published the systematic review protocol on PROSPERO (registration No. CRD42018084742) in March 2018 and performed the review according to PRISMA standards.^6^ The data that support the findings of this study are available from the corresponding author upon request.

We searched MEDLINE and EMBASE between 1990 and July 16, 2019 (Data Supplement) for original articles reporting prospective randomized controlled trials of cilostazol in patients with stroke, SVD, mild cognitive impairment, or dementia. We also searched clinical trial registries (www.isrctn.com; https://eudract.ema.europa.eu/; www.strokecenter.org/), conference proceedings, bibliographies of review papers, previous systematic reviews, and trial papers for relevant trials not identified in the search, and finally for secondary publications of included trials that might provide additional outcomes.

We included randomized, controlled, unconfounded, trials in patients with stroke, mild cognitive impairment or dementia, or radiological features of SVD, who were randomized to treatment with cilostazol. Control groups received placebo tablets, another antiplatelet, or received no cilostazol (open label). We excluded trials only published as conference abstracts, where translation into English was not possible, or where the full text was not available.

We included trials that reported any of the following: recurrent stroke (all, ischemic, hemorrhagic), incident dementia, incident mild cognitive impairment, change in cognitive test scores including domain specific scores, intracranial hemorrhage, other major/fatal bleeding, other systemic bleeding complications, death, myocardial infarction, dependency in activities of daily living, symptoms related to cilostazol use (such as nausea, headache, palpitations), change in white matter hyperintensities, progression/development of lacunes, microbleeds, perivascular spaces, brain atrophy (assessed by volume or validated score).

Two reviewers screened titles and abstracts of all identified articles (G.W. Blair, C. McHutchison), independently performed full text review of relevant papers, extracted data from included papers using standardized forms, and cross-checked their findings.

We extracted data on trial setting (hospital, community, etc), number of participants, sex, inclusion illness, diagnosis method including cognitive testing, proportion with lacunar stroke, randomization methods, time from onset of inclusion illness to randomization, blinding, treatment dose, duration, control allocation, concomitant antiplatelet or other agents, methods of outcome assessment, and proportion of patients with outcomes as listed above by intention to treat populations. We assessed study quality using the CONSORT (Consolidated Standards of Reporting Trials) criteria.^7^

Discrepancies between the 2 reviewers were resolved by discussion and a third reviewer (Dr Wardlaw) who cross-checked all data extraction.

### Meta-Analysis

We entered data into RevMan5 (version 5.3) software package. For most analyses, we grouped trials according to (1) their time to randomization (randomizing in acute/subacute versus later after stroke); and (2) use of other prescribed antiplatelet drug (none, cilostazol plus aspirin or clopidogrel versus aspirin or clopidogrel, cilostazol versus aspirin or clopidogrel) and meta-analyzed each outcome. We meta-analyzed symptoms by type. For death from all causes, we assumed no deaths in studies that did not report deaths. We used Peto odds ratio (OR) and 95% CIs for the meta-analyses, a preferred method where outcome events are infrequent.^8^

In exploratory sensitivity analyses, we ranked trials according to the proportion of patients with small vessel (lacunar) ischemic stroke, dichotomized into <40% and ≥40%, or unspecified. We also tested time from stroke to start of treatment and other antiplatelet drugs used.

We performed a meta-regression to test whether time to start treatment, proportion of patients with lacunar stroke, study duration, or comparison antiplatelet agent influenced the effect of cilostazol, using R version 3.6.2 (https://cran.r-project.org/) meta package.

We assessed risk of bias using funnel plots and heterogeneity using I^2^ and χ^2^ tests.

## Results

**Figure 1.**
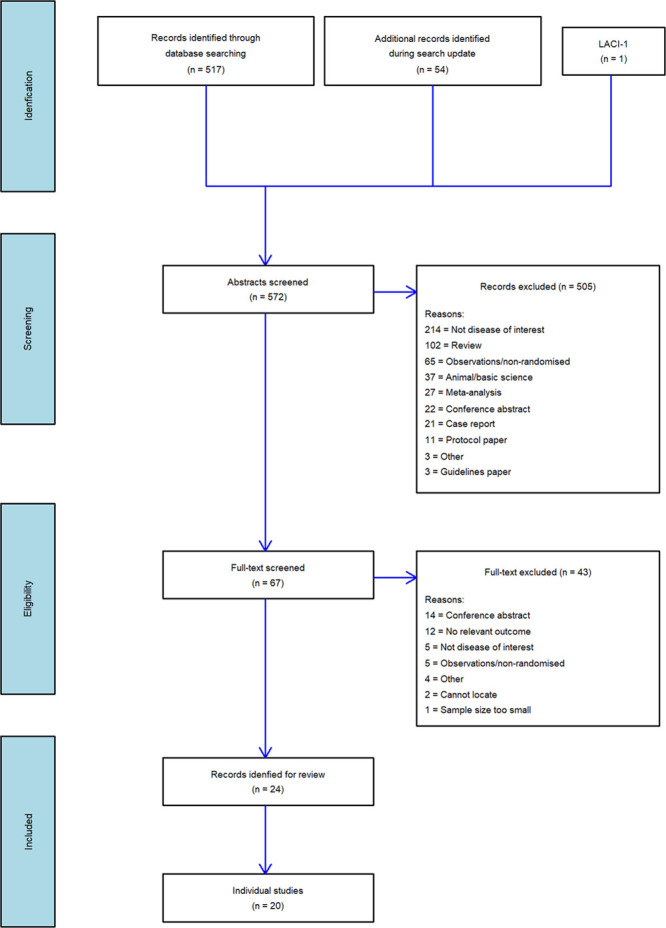
**PRISMA flow chart of study identification.**

We identified 572 articles but excluded 505 after abstract screening, and a further 43 after full text review (Figure 1). We included 20 unconfounded, original randomized controlled trials, published in 24 papers, including 10 505 participants (Table 1).

**Table 1.**
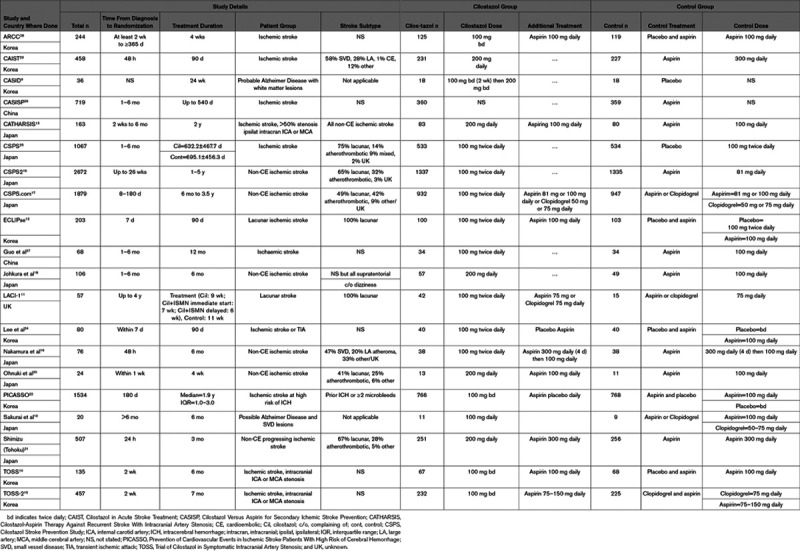
Characteristics of Included Studies

### Characteristics of Included Trials

The 20 trials had a median sample size of 183, range 20 to 2672. Eighteen trials included patients with stroke (n=10 449, Table 1) and 2 included patients with cognitive impairment or dementia of Alzheimer’s type and radiological evidence of SVD (n=56).^9,10^

Of the 18 trials in patients with stroke, 2 only included patients with lacunar stroke (n=515),^11,12^ 3 only included patients with intracranial artery stenosis (n=755),^13–15^ 6 only included patients with noncardioembolic ischemic stroke (n=5264),^16–21^ most trials excluded patients with cardioembolic stroke regardless of other inclusion criteria, and one trial included patients at high risk of intracerebral hemorrhage (n=1534).^22^ In 9/18 trials, the stroke was lacunar in ≥40% of participants (n=6943); in the other 9 trials, <40% of patients had a lacunar ischemic stroke or the subtype proportion was not specified (n=3262).

The time to randomization after diagnosis was <2 weeks in 8 (n=1940),^12,14,15,19–21,23,24^ between 2 weeks and 6 months in 5 (n=2123),^13,18,25–27^ and 6 months or later in 6 trials (n=6406; including the one trial in cognitive decline/dementia)^10,11,16,17,22,28^ and was not stated in the other trial in cognitive decline.^9^ The duration of trial treatment was 4 weeks in 3 (n=344),^19,20,28^ 10 weeks in 1 (n=57),^11^ 4 months in 4 (n=1236),^12,20,22,23^ 6 to 8 months in 5 (n=753; including both trials in cognitive decline/dementia),^9,10,14,15,18^ 12 months in 1 (n=68),^27^ and between 12 months and 5 years in 6 trials (n=8034).^13,16,17,22,25,26^

Eight trials used placebo tablets, the rest were open label (Table 1). One trial in stroke and one in Alzheimer’s disease tested cilostazol versus control in the absence of any other antiplatelet drug; 9 trials tested cilostazol plus aspirin or clopidogrel versus aspirin or clopidogrel; 8 trials tested cilostazol versus aspirin or clopidogrel, and 1 trial tested cilostazol plus aspirin versus clopidogrel plus aspirin.

Of the 18 trials that included patients with stroke, one^28^ did not record recurrent stroke outcomes, and one^10^ that included patients with cognitive impairment reported recurrent stroke; therefore, 18 trials provided data on recurrent stroke (all, ischemic, Table I in the Data Supplement). Sixteen trials reported recurrent hemorrhagic stroke, 18 reported death, 3 trials reported cognitive outcomes (2 trials in patients with cognitive impairment, one trial in stroke),^9–11^ 10 trials reported major cardiac outcomes, 7 assessed functional outcome (modified Rankin Scale) but only 5 gave results (precluding meta-analysis of effects of cilostazol on dependency), and about half the trials reported adverse symptoms (headache, nausea, palpitations, systemic bleeding; Table II in the Data Supplement). Outcomes are summarized in Table 2.

**Table 2.**
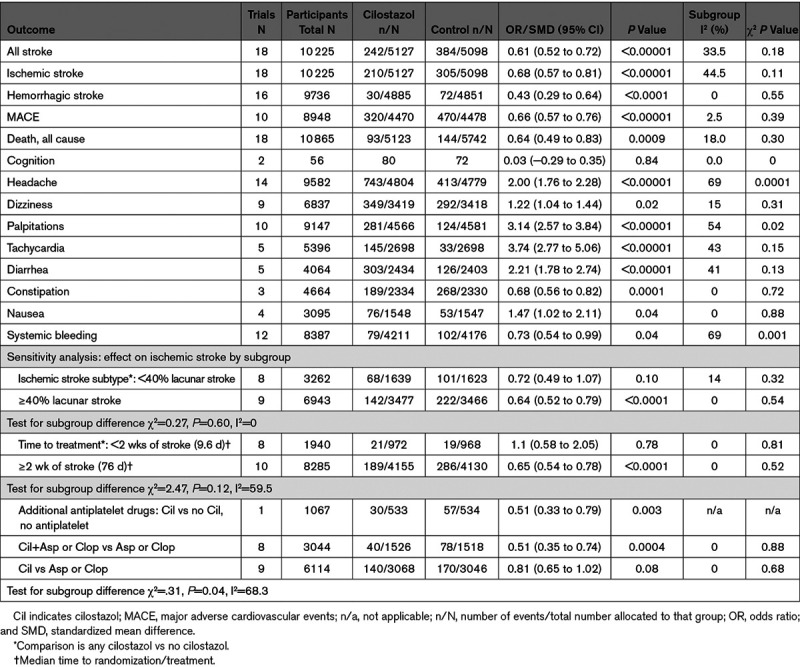
Summary of Main Results

### Recurrent Ischemic Stroke

**Figure 2.**
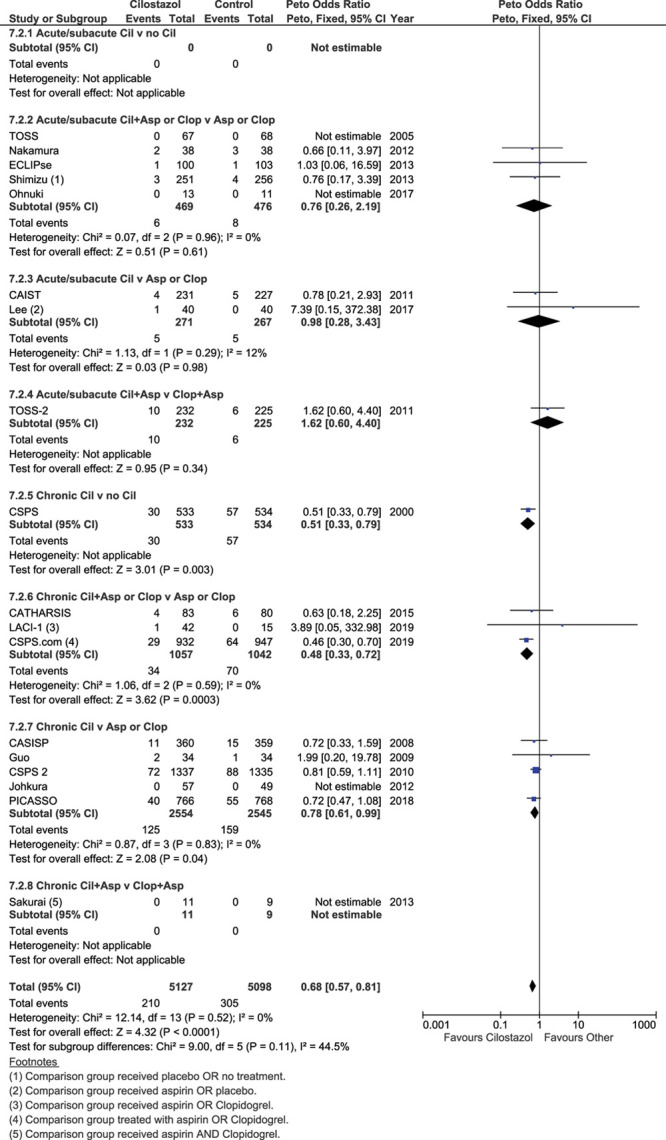
**Effect of cilostazol on ischemic stroke.** CAIST indicates Cilostazol in Acute Stroke Treatment; CSPS, Cilostazol Stroke Prevention Study; and TOSS, Trial of Cilostazol in Symptomatic Intracranial Artery.

Eighteen trials (n=10 225) reported recurrent ischemic stroke (cilostazol 5127, control 5098). Cilostazol decreased recurrent ischemic stroke (OR=0.68 [95% CI, 0.57–0.81]; *P*<0.0001), Figure 2, without heterogeneity. Most benefit appeared in the 9 trials testing cilostazol started >2 weeks after stroke (median 76 days; omitted in 3 trials) and given long term, where the ORs are all <1 regardless of comparator group or concomitant antiplatelet drug use (see sensitivity analyses below). In contrast, in the 8 trials starting cilostazol within 2 weeks of stroke (median 9.6 days; omitted in 4 trials) and assessing outcome at 1 to 4 months, the ORs all overlapped one, although the acute/subacute trials were smaller than the later-implementation/longer duration trials. A similar effect was seen for any recurrent stroke (18 trials, n=10 225, 5127 allocated cilostazol, 5098 allocated control) where cilostazol decreased the odds of any recurrent stroke (OR=0.61 [95% CI, 0.523–0.72]; *P*<0.00001), without heterogeneity (Figure I in the Data Supplement).

### Hemorrhagic Stroke

**Figure 3.**
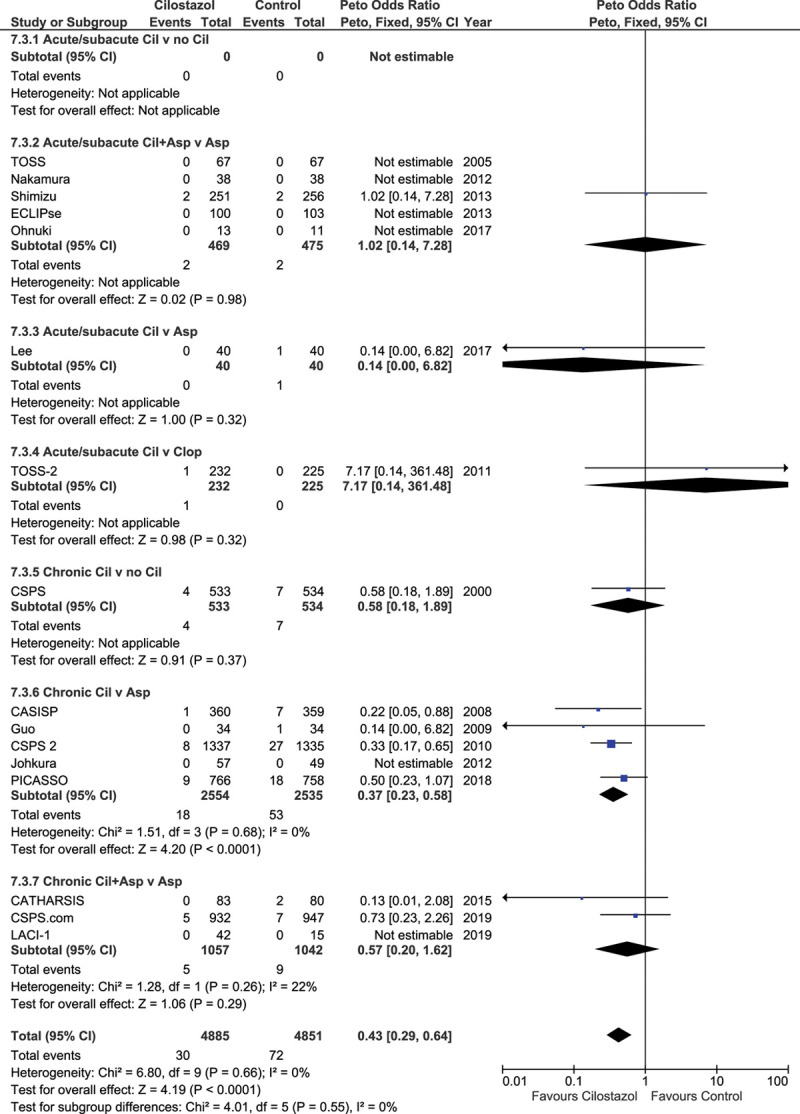
**Effect of cilostazol on hemorrhagic stroke.** CASISP indicates Cilostazol Versus Aspirin for Secondary Ichemic Stroke Prevention; CATHARSIS, Cilostazol-Aspirin Therapy Against Recurrent Stroke With Intracranial Artery Stenosis; and CSPS, Cilostazol Stroke Prevention Study.

Sixteen trials (n=9736) reported recurrent hemorrhagic stroke (cilostazol 4885, control 4851). Overall, cilostazol reduced hemorrhagic stroke (OR=0.43 [95% CI, 0.29–0.64]; *P*=0.0001), Figure 3, without heterogeneity. The pattern of effect was similar to that seen in all stroke and ischemic stroke although the reduced sample resulted in fewer individually significant results.

### Major Adverse Cardiovascular Events

Ten trials reported a composite outcome of major adverse cardiovascular events (cilostazol 4470, control 4478). Cilostazol decreased major adverse cardiovascular events (OR=0.66 [95% CI, 0.57–0.76]; *P*<0.00001), without heterogeneity (Figure II in the Data Supplement). Most benefit occurred in trials testing long-term cilostazol starting 6 months or more after stroke, where summary ORs are <1 regardless of whether cilostazol was compared with placebo or aspirin or of concomitant antiplatelet drug use.

### Death

Eighteen trials reported death from any cause (cilostazol 5123, control 5742). Overall, cilostazol decreased the odds of death (OR=0.64 [95% CI, 0.49–0.83]; *P*=0.0009), Figure III in the Data Supplement, without heterogeneity. Most benefit occurred in trials randomizing patients late after diagnosis while trials randomizing soon after stroke were more equivocal.

### Cognition

Two trials provided meta-analyzable results (cilostazol 29, control 27; Figure IV in the Data Supplement), but data were too sparse to draw conclusions. One trial (LACI-1) that could not be meta-analyzed reported a mean difference (adjusted for baseline) in Trail Making Test A of −4.0 (−12.7 to 4.7; *P*=0.37).

### Radiological Markers of SVD

Only 3 trials reported SVD imaging markers although each reported a different measure (silent infarcts, new ischemic lesion, microbleeds). Overall 55/557 participants allocated cilostazol developed an imaging lesion compared with 48/581 allocated control (OR=1.22 [95% CI, 0.81–1.84]; *P*=0.34).

### Adverse Symptoms

The types of symptoms reported by each study varied (Table II in the Data Supplement). In general, patients allocated cilostazol had more headache, dizziness, palpitations, tachycardia and diarrhea, but less constipation and nonstroke bleeding events (Table 2; Figure V in the Data Supplement). There was no heterogeneity for the above outcomes apart from systemic bleeding and palpitations (palpitations I^2^=54%, χ^2^=19.43, *P*=0.02; systemic bleeding I^2^=69%, χ^2^=25.6, *P*=0.001).

### Sensitivity Analyses

#### Lacunar Versus Nonlacunar Stroke

In the 8 trials with <40% or unstated proportion of patients with lacunar stroke (cilostazol 1639, control 1623), cilostazol did not reduce recurrent ischemic stroke (OR=0.72 [95% CI, 0.49–1.07]; *P*=0.10, without heterogeneity), Figure VIA in the Data Supplement. In the 9 trials with 40% or more patients with lacunar stroke (cilostazol 3477, control 3466; of which, 6 trials, total n=4964, included 58% or more lacunar strokes), cilostazol reduced recurrent ischemic stroke (OR=0.64 [95% CI, 0.52–0.79]; *P*<0.0001, without heterogeneity). However, the effect of cilostazol on recurrent ischemic stroke did not differ between the 2 subgroups (<40% or ≥40% with lacunar stroke), on formal testing (χ^2^ for difference=0.27, *P*=0.60, I^2^=0%, *P*=0.60, without heterogeneity).

#### Time From Stroke to Treatment

Patients allocated treatment within 2 weeks of stroke, and where treatment was generally continued for no more than 4 months, those allocated cilostazol had similar rates of recurrent ischemic stroke (21/972) than those allocated control (19/968), OR=1.10 (95% CI, 0.58–2.05), *P*=0.78 without heterogeneity (Figure VIB in the Data Supplement). In patients starting treatment beyond 2 weeks after stroke (median), and where treatment was generally continued for 6 months to 5 years, those allocated to cilostazol had fewer recurrent ischemic strokes (189/4155) than those allocated control (286/4130), OR=0.65 (95% CI, 0.54–0.78), *P*<0.00001, without heterogeneity. However, there was no evidence of a between group difference (acute versus late, χ^2^ 2.47, *P*=0.12, with moderate heterogeneity, I^2^=59.5%).

#### Concomitant Antiplatelet Drugs

Trials which randomized between cilostazol and no cilostazol in the absence or presence of concomitant aspirin or clopidogrel showed similar benefit for cilostazol (no aspirin, OR=0.51 [95% CI, 0.33–0.79]; *P*=0.003; all patients received aspirin or clopidogrel, OR=0.51 [95% CI, 0.35–0.74]; *P*=0.0004) (Figure VIC in the Data Supplement). However, in trials where cilostazol was compared with aspirin or clopidogrel, including one trial randomizing to cilostazol+aspirin versus clopidogrel+aspirin,^15^ there was no definite benefit of cilostazol (OR=0.81 [95% CI, 0.65–1.02]; *P*=0.08). Across the 3 subgroups, there was evidence of between-subgroup differences (χ^2^, 6.31; *P*=0.04), and moderate heterogeneity (I^2^=68.3%). Restricting the analysis to trials comparing cilostazol with one antiplatelet drug in the absence of another antiplatelet drug by excluding the TOSS2 trial showed benefit of cilostazol over the other antiplatelet drug (OR=0.78 [95% CI, 0.62–0.99]; *P*=0.04, without heterogeneity) and removed the evidence of between-subgroup difference (χ2, 5.19; *P*=0.07), but retained heterogeneity (I^2^=61.4%).

#### Meta-Regression

Meta-regression of time to treatment, duration of treatment, and proportion of lacunar strokes, adjusted for comparator antiplatelet agent, did not identify any significant subgroup effects on outcomes of recurrent ischemic or hemorrhagic stroke.

### Sources of Bias

The median trial quality was 23.5/37 (minimum 14, maximum 35), with methods sections attaining the lowest scores on average (Table III and Figure VII in the Data Supplement).

Funnel plots on all stroke and ischemic stroke showed some skew suggesting reporting bias but not for hemorrhagic stroke did not show any skew (Figure VIII in the Data Supplement).

## Discussion

Cilostazol reduced recurrent stroke, recurrent ischemic stroke, recurrent hemorrhagic stroke, death and major adverse cardiovascular events compared with control, in the presence or absence of aspirin, or when compared directly with aspirin (data were limited for comparison with clopidogrel). Most benefit occurred in trials that randomized patients at 2 or more weeks after stroke and administered cilostazol for at least 6 months or longer, without evidence of increased risk with long-term treatment. There were very few data on the effect of cilostazol on functional outcome, cognitive decline, or radiological markers of SVD. Adverse symptoms such as headache, palpitations, dizziness, and diarrhea were clearly increased with cilostazol although, importantly, systemic bleeding events were reduced.

The review limitations are related to the available data and include variation between trials in antiplatelet drug use, times to randomization after stroke, durations of treatment, not reporting dependency outcomes, and lack of information on stroke subtypes. Included studies varied greatly in sample size and some studies had no events in either group for certain outcomes. Antiplatelet therapy has changed since some studies were completed. Guidelines now advice dual antiplatelets short term after transient ischemic attack or minor ischemic stroke, followed by clopidogrel longer term. Only one study compared cilostazol to clopidogrel and both groups also received aspirin.^15^ Only 2 trials recruited patients with cognitive presentations and only one trial in stroke assessed cognition. The median trial quality was moderate (23.5/37). Thus, despite the total available data from trials of cilostazol totaling over 10 000 patients, the conclusions have limitations. There were also strengths of the review, including prospective protocol registration, assessment of methodological quality, double assessment of papers and data extraction, and careful harmonization of the trials for analysis.

Cilostazol may have more benefit on several outcomes where participants were randomized later after stroke. Although arbitrary, the trials naturally dichotomized into those randomizing within 2 weeks of stroke and those randomizing at >2 weeks after stroke, of which about a third randomized between 2 weeks and 6 months and 2 thirds randomized after 6 months. Trials randomizing >6 months after stroke had long durations of treatment and follow-up. Thus, the apparent benefit of cilostazol in trials randomizing late rather than early may reflect the paucity of acute trials, shorter duration of treatment, higher proportion of lacunar strokes, or that cilostazol is less effective in preventing early recurrent stroke. Similar results have been seen with another phosphodiesterase inhibitor dipyridamole (PDE5 inhibitor) with mild-antiplatelet and proendothelial effects,^2^ which reduced stroke recurrence while increasing headache, mostly in Western populations. The risk of stroke recurrence varies by stroke subtype, atherothromboembolic stroke recurrence risk being the highest immediately after transient ischemic attack/minor stroke, then declining, whereas lacunar stroke has lower risk of early recurrence but the rate remains elevated in the longer term.

Cilostazol’s apparent greater benefit late after stroke could reflect several possible mechanisms. Weaker antiplatelet effects^3^ and hence inferior stroke prevention compared with aspirin or clopidogrel early after transient ischemic attack/stroke (when stronger antiplatelet activity may be more beneficial) is supported by the neutral effect of cilostazol on ischemic stroke recurrence compared with aspirin or clopidogrel (Figure VIC in the Data Supplement). Increasing benefit of cilostazol late after stroke was also demonstrated in CASISP, which found no difference in recurrent stroke between cilostazol and aspirin within 6 months of stroke, but increasing benefit of cilostazol versus aspirin thereafter.^26^ The increased benefit of cilostazol later after stroke may reflect that its mechanisms of action are more relevant to lacunar stroke where recurrence occurs late, supported by increased benefit in trials including more patients with lacunar stroke (Figure VIA in the Data Supplement). Potential benefits for lacunar stroke include endothelial stabilization, improved myelin repair, and better astrocyte-to-neuronal energy supply,^2,11^ all of which may take some time to accrue. The lower cerebral and systemic hemorrhage risks would also confer benefit over other antiplatelet drugs, which typically have higher bleeding risk the longer they are given, a reason for early stopping of the SPS3 Trial (dual versus single antiplatelet drugs) for lacunar stroke^29^ and seen in the present meta-analysis even in the presence of other antiplatelet drugs. The PICASSO (PreventIon of CArdiovascular events in iSchemic Stroke patients with high risk of cerebral hemOrrhage) trial suggests that the benefits of cilostazol may extend to reducing recurrent stroke and systemic bleeding even in patients at high risk of intracerebral hemorrhage.^22^

More data are needed to overcome the limitations of the current data, to determine the effect of cilostazol on functional and cognitive outcomes after stroke, and on delaying cognitive decline. If the effects of cilostazol seen in laboratory models translate to people (myelin repair, improved neuronal energy supply, and endothelial stabilization) and help to prevent progression of brain injury, then cilostazol might also prevent physical decline seen in SVD. Future studies should compare cilostazol to modern antiplatelet regimes, stratify patients by stroke or cognitive impairment, provide more data on cognitive, imaging and functional outcomes, and on tolerability and compliance. Several ongoing studies address these issues. LACI-2 (ISRCTN 14911850) is assessing cilostazol long-term after lacunar ischemic stroke in the UK including 1-year cognitive and brain magnetic resonance imaging follow-up (target n=400). The COMCID trial (Asia-Pacific) is assessing cilostazol’s effects on cognitive function, incident dementia, and hippocampal volumes (NCT02491268). Other trials are assessing short-term effects of cilostazol on cerebrovascular reactivity (eg, Oxford Hemodynamic Adaptation to Reduce Pulsatility Trial [OxHARP], NCT03855332, target n=76).

Cilostazol shows promise for ischemic stroke prevention, with lower risk of hemorrhagic complications, particularly long term. Its place in stroke therapy may be in chronic secondary prevention rather than the acute phase. However, most data are from Asia Pacific countries where stroke etiologies and other factors may differ from other world regions, hence the need for more data. Despite its encouraging safety profile (lower bleeding risk and death), cilostazol causes several symptoms (headache, palpitations, diarrhea, nausea), which may limit tolerance, requiring more data to guide future routine use. It is licenced in Europe and the Americas for treatment of symptomatic peripheral vascular disease and stroke prevention where other antiplatelet agents have failed or are not tolerated. However, more evidence is needed before it is used more widely in stroke in routine practice.

## Acknowledgments

C. McHutchison performed article search and data extraction, meta-analysis, drafting, and review of manuscript. G.W. Blair performed protocol design, article search and data extraction, analysis, drafting, and review of manuscript. Dr Appleton performed review of protocol and manuscript. P.M. Bath performed protocol design, and review of manuscript. Dr Wardlaw performed review conception, protocol design, supervision, article search, data extraction, analysis, drafting, review of manuscript, and approval of the final manuscript for submission.

## Sources of Funding

Chest Heart Stroke Scotland (Res 14/A157); European Union Horizon 2020 ‘SVDs@Target’ (No. 666881); Fondation Leducq Transatlantic Network on Perivascular Spaces in SVD (16CVD05); UK Dementia Research Institute (DRI Ltd, UK Medical Research Council, Alzheimer’s Society, Alzheimer’s Research UK); Alzheimer’s Society (AS-PG-14-033); Stroke Association (Princess Margaret Research Development Fellowship, Stroke Association-Garfield Weston Foundation Senior Clinical Lectureship); NHS Research Scotland; National Institute of Health Research; British Heart Foundation (CS/15/5/31475).

## Disclosures

Dr Wardlaw, P.M. Bath, G.W. Blair, Dr Appleton, and Dr Doubal worked on the LACI-1 and LACI-2 trials testing cilostazol in lacunar stroke. P.M. Bath led systematic reviews of dipyridamole. The other authors report no conflicts.

## Supplementary Material


