# Supplementary material for: Cilostazol for Secondary Prevention of Stroke and Cognitive Decline: Systematic Review and Meta-Analysis
Source: Stroke. 2020 Jul 10;51(8):2374–85. doi: 10.1161/STROKEAHA.120.029454 (PMC7382534; doi:10.1161/STROKEAHA.120.029454)
Supplement: Supplementary file 1 [file str-51-2374-s001.pdf]

**Supplemental Material**

Cilostazol for secondary prevention of stroke and cognitive decline: Systematic review and meta-analysis

Caroline McHutchison, MSc, [1]
Gordon W Blair, MRCP(UK), [1]
Jason P. Appleton, MCRP, PhD,[2,3]
Francesca M Chappell, PhD,[1]
Fergus Doubal, PhD, FRCP,[1]
Philip M Bath, FMedSci, [2]
Joanna M Wardlaw, MD, FMedSci[1]

**Contents**

Search Strategy

Table I Outcomes reported

Table II Adverse symptoms reported

Table III Quality scores on CONSORT criteria

Figure I: All Stroke recurrence forest plot

Figure II: Major adverse cardiovascular events forest plot

Figure III: Death (all cause) forest plot

Figure IV: Cognition

Figure V: Adverse symptoms including systemic bleeding

Figure VI: Sensitivity analyses – Proportion of lacunar stroke, time to treatment, use of antiplatelet drugs

Figure VII: Chart of proportions of papers meeting CONSORT quality criteria

Figure VIII: Funnel plot data on Ischaemic stroke

**Search Strategy**

1) brain isch?emia/ or brain infarction/ or cerebral infarction/ or hypoxia-isch?emia, brain/ or stroke$.tw.
2) cerebral vascular attack/ or cerebrovascular attack/ or cerebral vascular accident/ or cerebrovascular accident/ or CVA.tw.
3) (lacun$ or small vessel$ or small infarct$ or microinfarct$ or subcortical lesion$ or subcortical infarct$).tw.
4) leukoaraiosis/ or Binswanger’s disease.tw.
5) (white matter hyperintensit$ or WMH).tw.
6) (small vessel disease or SVD).tw.
7) (white matter lesion$ or WML).tw.
8) vascular dementia.tw
9) dementia.tw
10) Alzheimer’s disease/ Alzheimer$.tw
11) mild cognitive impairment / or MCI/ or cognitive impairment.tw
12) 1 or 2 or 3 or 4 or 5 or 6 or 7 or 8 or 9 or 10 or 11
13) cilostazol.tw.
14) pletal.tw.
15) 13 or 14
16) 12 AND 15
17) limit 16 to humans
18) remove duplicates from 17

**Supplement Table I:** Outcomes reported by each study

| Study | Outcome | | | | | | | | |
|---|---|---|---|---|---|---|---|---|---|
| | Stroke reoccurrence | | | MACE | Adverse symptoms [$] | Functional outcome (mRS) | Death* | Cognition | Radiological findings |
| | All stroke | Ischaemic stroke | Haemorrhagic stroke | | | | | | |
| **ARCC[1]** | | | | | ✓ | | | | |
| **CAIST[2]** | ✓ | ✓ | | ✓ | ✓ | ✓ | ✓ | | |
| **CASID[3]** | | | | | ✓ | | | ✓ | |
| **CASISP[4]** | ✓ | ✓ | ✓ | | ✓ | ✓[#] | ✓ | | ✓ |
| **CATHARSIS[5]** | ✓ | ✓ | ✓ | ✓ | ✓ | ✓ | ✓ | | ✓ |
| **CSPS[6-8]** | ✓ | ✓ | ✓ | ✓ | ✓ | | ✓ | | |
| **CSPS2[9-11]** | ✓ | ✓ | ✓ | ✓ | ✓ | | ✓ | | |
| **CSPS.com[12, 13]** | ✓ | ✓ | ✓ | ✓ | ✓ | ✓[#] | ✓ | | |
| **ECLIPse[14, 15]** | ✓ | ✓ | ✓ | | ✓ | | ✓ | | |
| **Guo[16]** | ✓ | ✓ | ✓ | | ✓ | ✓[#] | ✓ | | |
| **Johkura[17]** | ✓ | ✓ | ✓ | | ✓ | | ✓ | | |
| **LACI-1[18-20]** | ✓ | ✓ | ✓ | | ✓ | | ✓ | ✓ | ✓ |
| **Lee[21]** | ✓ | ✓ | ✓ | | ✓ | | ✓ | | |
| **Nakamura[22]** | ✓ | ✓ | ✓ | ✓ | ✓ | ✓ | ✓ | | |
| **Ohnuki[23]** | ✓ | ✓ | ✓ | | ✓ | | ✓ | | |
| **PICASSO[24-26]** | ✓ | ✓ | ✓ | ✓ | ✓ | | ✓ | | |
| **Sakurai[27]** | ✓ | ✓ | | | | | ✓ | ✓ | |
| **Shimizu[28]** | ✓ | ✓ | ✓ | ✓ | ✓ | ✓ | ✓ | | |
| **TOSS[29]** | ✓ | ✓ | ✓ | ✓ | | | ✓ | | |
| **TOSS-2[30]** | ✓ | ✓ | ✓ | ✓ | ✓ | | ✓ | | ✓ |
| **TOTAL** | **18** | **18** | **16** | **10** | | | **18** | **3** | **4** |

[#] = not reported; [$] = see Supplementary Table II for details of adverse symptoms; mRS = modified Rankin Scale

**Supplement Table II:** Adverse symptoms reported by each study (references are given in Supplement Table I)

| Study | Outcome | | | | | | | |
|---|---|---|---|---|---|---|---|---|
| | Adverse events | | | | | | | |
| | Headaches | Palpitations | Dizziness | Nausea | Constipation | Diarrhoea | Tachycardia | Bleeding |
| ARCC | ✓ | | | | | | | ✓ |
| CAIST | ✓ | ✓ | ✓ | ✓ | ✓ | ✓ | | |
| CASID | ✓ | | ✓ | ✓ | | ✓ | | |
| CASISP | ✓ | ✓ | ✓ | | | | ✓ | ✓ |
| CATHARSIS | | | | | | | | |
| CSPS | ✓ | ✓ | ✓ | ✓ | | | | |
| CSPS2 | ✓ | ✓ | ✓ | | ✓ | ✓ | ✓ | ✓ |
| CSPS.com | ✓ | ✓ | | | | | ✓ | ✓ |
| ECLIPse | ✓ | ✓ | ✓ | | | | | |
| Guo | ✓ | | ✓ | | | | ✓ | ✓ |
| Johkura | ✓ | | | | | | ✓ | ✓ |
| LACI-1 | ✓ | | | | | | | ✓ |
| Lee | ✓ | ✓ | ✓ | | | ✓ | | |
| Nakamura | | ✓ | | | | | | ✓ |
| Ohnuki | | | | | | | | ✓ |
| PICASSO | ✓ | ✓ | ✓ | ✓ | ✓ | ✓ | | ✓ |
| Sakurai | | | | | | | | |
| Shimizu | ✓ | ✓ | | | | | | ✓ |
| TOSS | | | | | | | | |
| TOSS-2 | | | | | | | | ✓ |
| TOTAL | 14 | 10 | 9 | 4 | 3 | 5 | 5 | 12 |

**Supplement Table III.** Study Quality by CONSORT criteria section. CONSORT consists of 37 items rating the presence (yes/no) of information included in each study. Where criterion is met, a score of one for the item was given (possible scores ranged from 0 to 37). Overall study quality ranged from 14-35 (mean=24, SD=5.88).

| | **Number of items** | **Min** | **Max** | **Median** | **IQR** |
|---|---|---|---|---|---|
| Title & abstract | 2 | 0 | 2 | 2 | 1-2 |
| Introduction | 2 | 2 | 2 | 2 | 2-2 |
| Methods | 17 | 4 | 16 | 9.5 | 5-11.25 |
| Results | 10 | 3 | 10 | 8 | 6.75-9 |
| Discussion | 3 | 1 | 3 | 3 | 2-3 |
| Other information | 3 | 0 | 3 | 1.5 | 0-2 |
| ***TOTAL*** | ***37*** | ***14*** | ***35*** | ***23.5*** | ***20-27.5*** |

**Supplement Figure I.** Effect of cilostazol on recurrent stroke, all types.

| Study or Subgroup | Cilostazol Events | Cilostazol Total | Control Events | Control Total | Weight | Peto Odds Ratio Peto, Fixed, 95% CI | Year |
|---|---|---|---|---|---|---|---|
| **7.1.1 Acute/subacute Cil v no Cil** | | | | | | | |
| **Subtotal (95% CI)** | | **0** | | **0** | | **Not estimable** | |
| Total events | 0 | | 0 | | | | |
| Heterogeneity: Not applicable | | | | | | | |
| Test for overall effect: Not applicable | | | | | | | |
| **7.1.2 Acute/subacute Cil+Asp or Clop v Asp or Clop** | | | | | | | |
| TOSS | 0 | 67 | 0 | 68 | | Not estimable | 2005 |
| Nakamura (1) | 2 | 38 | 3 | 38 | 0.8% | 0.66 [0.11, 3.97] | 2012 |
| Shimizu (2) | 5 | 251 | 6 | 256 | 1.9% | 0.85 [0.26, 2.80] | 2013 |
| ECLIPse | 1 | 100 | 1 | 103 | 0.3% | 1.03 [0.06, 16.59] | 2013 |
| Ohnuki | 0 | 13 | 0 | 11 | | Not estimable | 2017 |
| **Subtotal (95% CI)** | | **469** | | **476** | **3.0%** | **0.81 [0.32, 2.06]** | |
| Total events | 8 | | 10 | | | | |
| Heterogeneity: Chi² = 0.09, df = 2 (P = 0.96); I² = 0% | | | | | | | |
| Test for overall effect: Z = 0.45 (P = 0.66) | | | | | | | |
| **7.1.3 Acute/subacute Cil v Asp or Clop** | | | | | | | |
| CAIST | 5 | 231 | 9 | 227 | 2.3% | 0.55 [0.19, 1.58] | 2011 |
| Lee (3) | 1 | 40 | 1 | 40 | 0.3% | 1.00 [0.06, 16.27] | 2017 |
| **Subtotal (95% CI)** | | **271** | | **267** | **2.7%** | **0.59 [0.22, 1.59]** | |
| Total events | 6 | | 10 | | | | |
| Heterogeneity: Chi² = 0.16, df = 1 (P = 0.69); I² = 0% | | | | | | | |
| Test for overall effect: Z = 1.04 (P = 0.30) | | | | | | | |
| **7.1.4 Acute/subacute Cil+Asp v Clop+Asp** | | | | | | | |
| TOSS-2 | 11 | 232 | 6 | 225 | 2.8% | 1.78 [0.68, 4.69] | 2011 |
| **Subtotal (95% CI)** | | **232** | | **225** | **2.8%** | **1.78 [0.68, 4.69]** | |
| Total events | 11 | | 6 | | | | |
| Heterogeneity: Not applicable | | | | | | | |
| Test for overall effect: Z = 1.17 (P = 0.24) | | | | | | | |
| **7.1.5 Chronic Cil v no Cil** | | | | | | | |
| CSPS | 34 | 533 | 64 | 534 | 15.3% | 0.51 [0.34, 0.77] | 2006 |
| **Subtotal (95% CI)** | | **533** | | **534** | **15.3%** | **0.51 [0.34, 0.77]** | |
| Total events | 34 | | 64 | | | | |
| Heterogeneity: Not applicable | | | | | | | |
| Test for overall effect: Z = 3.17 (P = 0.002) | | | | | | | |
| **7.1.6 Chronic Cil+Asp or Clop v Pla+Asp or Clop** | | | | | | | |
| CATHARSIS | 4 | 83 | 8 | 80 | 1.9% | 0.47 [0.15, 1.52] | 2015 |
| CSPS.com (4) | 34 | 932 | 71 | 947 | 17.0% | 0.48 [0.33, 0.71] | 2019 |
| LACI-1 (5) | 1 | 42 | 0 | 15 | 0.1% | 3.89 [0.05, 332.98] | 2019 |
| **Subtotal (95% CI)** | | **1057** | | **1042** | **19.1%** | **0.49 [0.34, 0.71]** | |
| Total events | 39 | | 79 | | | | |
| Heterogeneity: Chi² = 0.84, df = 2 (P = 0.66); I² = 0% | | | | | | | |
| Test for overall effect: Z = 3.78 (P = 0.0002) | | | | | | | |
| **7.1.7 Chronic Cil v Asp or Clop** | | | | | | | |
| CASISP | 12 | 360 | 22 | 359 | 5.6% | 0.54 [0.27, 1.07] | 2008 |
| Guo (6) | 2 | 34 | 1 | 34 | 0.5% | 1.99 [0.20, 19.78] | 2009 |
| CSPS 2 | 82 | 1337 | 119 | 1335 | 31.9% | 0.67 [0.50, 0.89] | 2010 |
| Johkura | 0 | 57 | 0 | 49 | | Not estimable | 2012 |
| Sakurai (7) | 0 | 11 | 0 | 9 | | Not estimable | 2013 |
| PICASSO | 48 | 766 | 73 | 768 | 19.1% | 0.64 [0.44, 0.93] | 2018 |
| **Subtotal (95% CI)** | | **2565** | | **2554** | **57.1%** | **0.65 [0.53, 0.81]** | |
| Total events | 144 | | 215 | | | | |
| Heterogeneity: Chi² = 1.25, df = 3 (P = 0.74); I² = 0% | | | | | | | |
| Test for overall effect: Z = 3.89 (P < 0.0001) | | | | | | | |
| **Total (95% CI)** | | **5127** | | **5098** | **100.0%** | **0.61 [0.52, 0.72]** | |
| Total events | 242 | | 384 | | | | |
| Heterogeneity: Chi² = 9.85, df = 13 (P = 0.71); I² = 0% | | | | | | | |
| Test for overall effect: Z = 5.89 (P < 0.00001) | | | | | | | |
| Test for subgroup differences: Chi² = 7.52, df = 5 (P = 0.18), I² = 33.5% | | | | | | | |

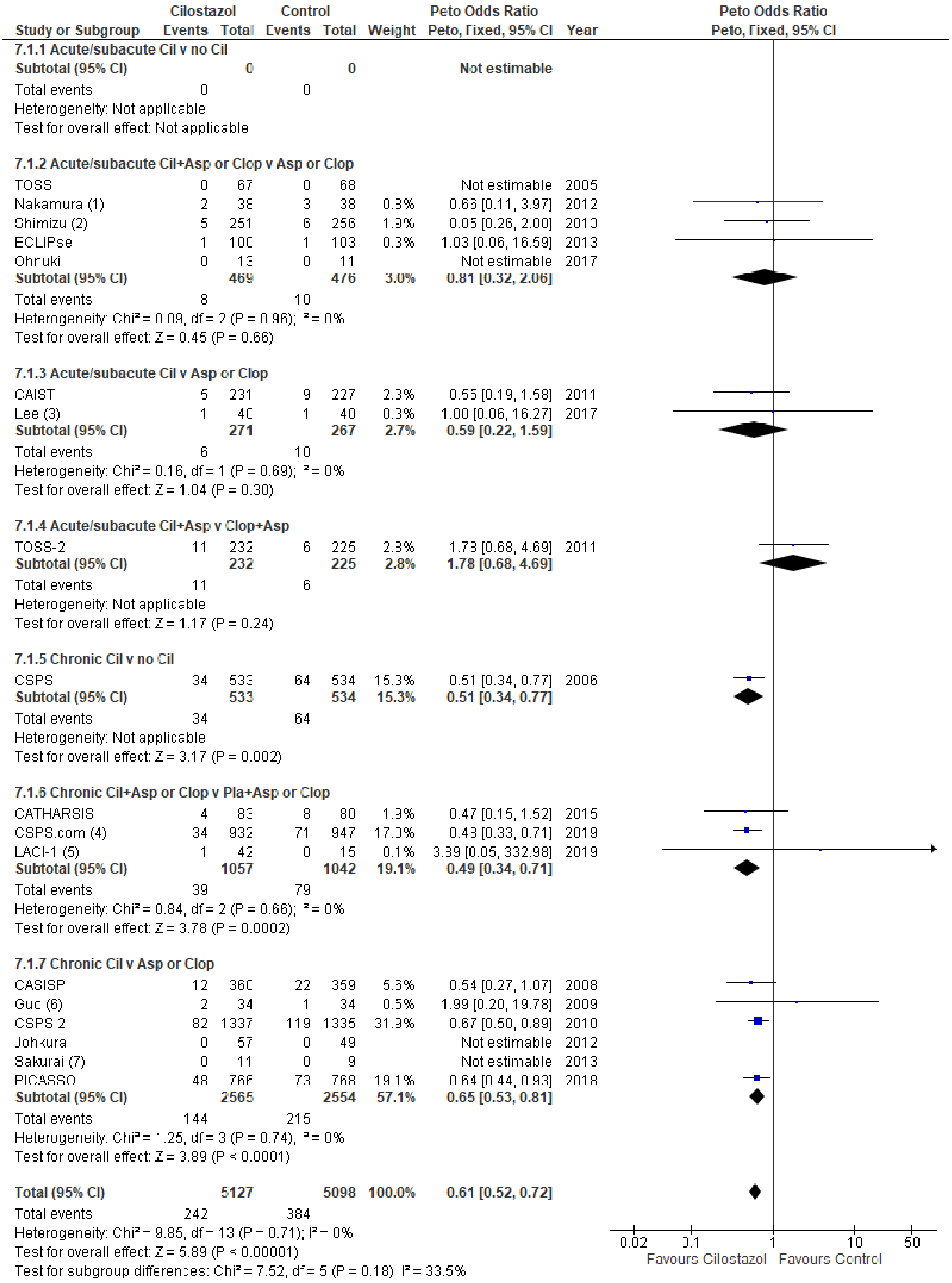


Footnotes
(1) Cilostazol group received aspirin AND Cilostazol.
(2) Comparison group received placebo OR no treatment.
(3) Comparison group received aspirin OR placebo.
(4) Comparison group treated with aspirin OR Clopidogrel.
(5) Comparison group received aspirin OR Clopidogrel.
(6) Includes TIA.
(7) Comparison group received aspirin AND Clopidogrel.

**Supplement Figure II** Effect of cilostazol on Major Adverse Cardiovascular Events (MACE)

| Study or Subgroup | Cilostazol Events | Cilostazol Total | Control Events | Control Total | Weight | Peto Odds Ratio Peto, Fixed, 95% CI | Year |
|---|---|---|---|---|---|---|---|
| **7.5.1 Acute/subacute Cil v Pla** | | | | | | | |
| TOSS (1) | 2 | 67 | 2 | 68 | 0.5% | 1.02 [0.14, 7.37] | 2005 |
| Shimizu (2) | 5 | 251 | 8 | 256 | 1.8% | 0.64 [0.21, 1.91] | 2013 |
| **Subtotal (95% CI)** | | **318** | | **324** | **2.3%** | **0.71 [0.27, 1.86]** | |
| Total events | 7 | | 10 | | | | |
| Heterogeneity: Chi² = 0.16, df = 1 (P = 0.69); I² = 0% | | | | | | | |
| Test for overall effect: Z = 0.70 (P = 0.49) | | | | | | | |
| **7.5.2 Acute/subacute Cil v Asp** | | | | | | | |
| CAIST (3) | 6 | 231 | 9 | 227 | 2.0% | 0.65 [0.23, 1.82] | 2011 |
| Nakamura (4) | 0 | 38 | 1 | 38 | 0.1% | 0.14 [0.00, 6.82] | 2012 |
| **Subtotal (95% CI)** | | **269** | | **265** | **2.2%** | **0.59 [0.22, 1.59]** | |
| Total events | 6 | | 10 | | | | |
| Heterogeneity: Chi² = 0.58, df = 1 (P = 0.45); I² = 0% | | | | | | | |
| Test for overall effect: Z = 1.05 (P = 0.29) | | | | | | | |
| **7.5.3 Acute/Subacute Cil v Clop** | | | | | | | |
| TOSS-2 (5) | 15 | 232 | 10 | 225 | 3.3% | 1.48 [0.66, 3.31] | 2011 |
| **Subtotal (95% CI)** | | **232** | | **225** | **3.3%** | **1.48 [0.66, 3.31]** | |
| Total events | 15 | | 10 | | | | |
| Heterogeneity: Not applicable | | | | | | | |
| Test for overall effect: Z = 0.95 (P = 0.34) | | | | | | | |
| **7.5.4 Chronic Cil v Pla** | | | | | | | |
| CSPS (6) | 48 | 533 | 73 | 534 | 15.1% | 0.63 [0.43, 0.92] | 2006 |
| **Subtotal (95% CI)** | | **533** | | **534** | **15.1%** | **0.63 [0.43, 0.92]** | |
| Total events | 48 | | 73 | | | | |
| Heterogeneity: Not applicable | | | | | | | |
| Test for overall effect: Z = 2.40 (P = 0.02) | | | | | | | |
| **7.5.5 Chronic Cil v Asp** | | | | | | | |
| CSPS 2 (7) | 138 | 1337 | 186 | 1335 | 40.0% | 0.71 [0.56, 0.90] | 2010 |
| CATHARSIS (8) | 5 | 83 | 11 | 80 | 2.0% | 0.42 [0.15, 1.18] | 2015 |
| PICASSO (9) | 63 | 766 | 80 | 768 | 18.2% | 0.77 [0.55, 1.09] | 2018 |
| CSPS.com (10) | 38 | 932 | 90 | 947 | 16.8% | 0.43 [0.30, 0.61] | 2019 |
| **Subtotal (95% CI)** | | **3118** | | **3130** | **77.1%** | **0.64 [0.54, 0.76]** | |
| Total events | 244 | | 367 | | | | |
| Heterogeneity: Chi² = 7.57, df = 3 (P = 0.06); I² = 60% | | | | | | | |
| Test for overall effect: Z = 5.22 (P < 0.00001) | | | | | | | |
| **Total (95% CI)** | | **4470** | | **4478** | **100.0%** | **0.66 [0.57, 0.76]** | |
| Total events | 320 | | 470 | | | | |
| Heterogeneity: Chi² = 12.41, df = 9 (P = 0.19); I² = 27% | | | | | | | |
| Test for overall effect: Z = 5.61 (P < 0.00001) | | | | | | | |
| Test for subgroup differences: Chi² = 4.10, df = 4 (P = 0.39), I² = 2.5% | | | | | | | |

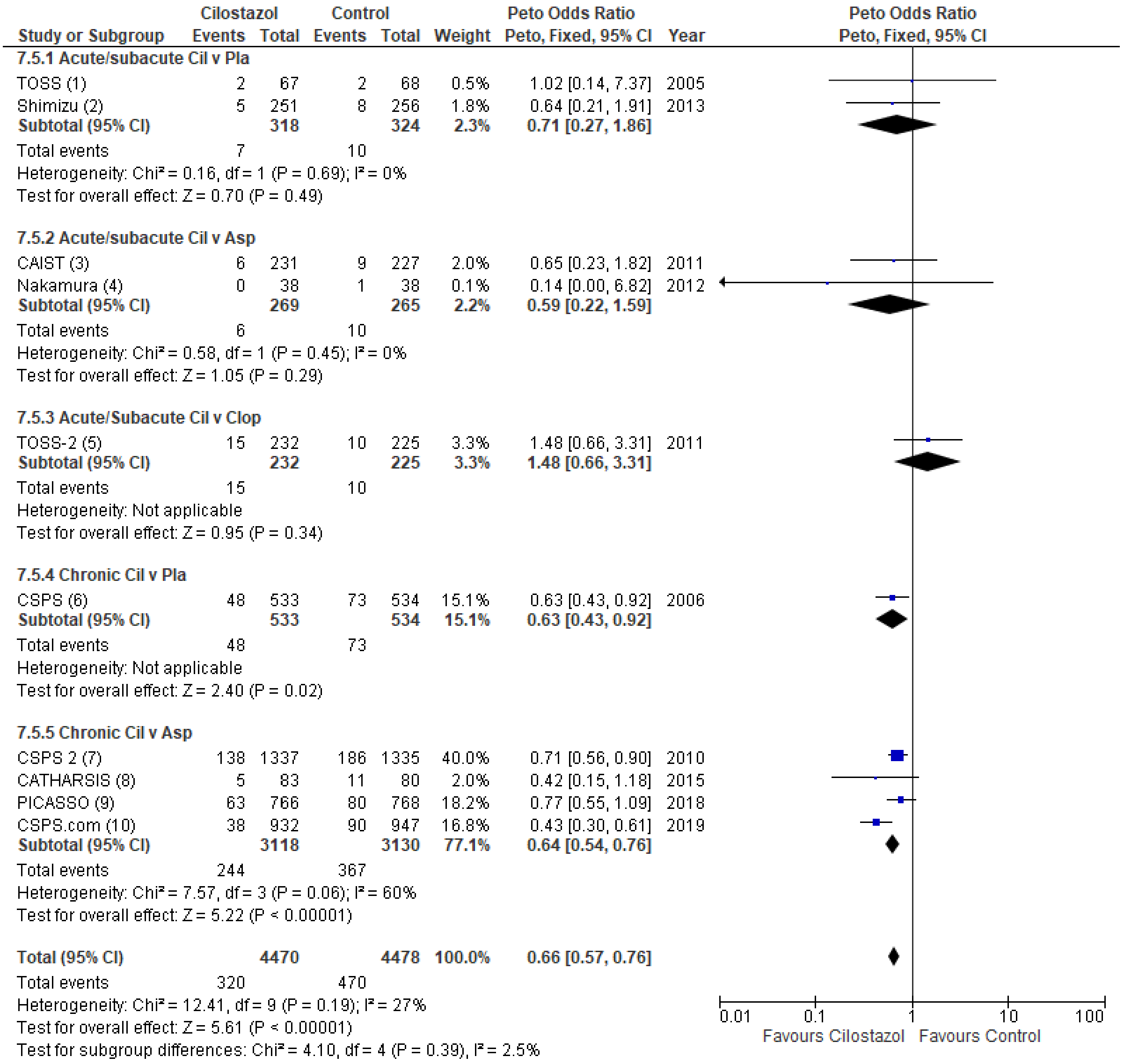


Footnotes

(1) Includes acute coronary events.
(2) Includes cerebral infarction, intracranial haemorrhage and congestive heart failure. Comparison group received placebo OR no treatment.
(3) Includes non-fatal stroke, non-fatal MI, vascular death or other cardiovascular events requiring hospitalisation.
(4) Cilostazol group received aspirin AND Cilostazol.
(5) Includes non-fatal stroke, non-fatal MI and vascular death.
(6) Includes cerebral infarction, TIA, MI, intracranial hemorrhage and other vascular events.
(7) Includes cerebral infarction, ischaemic cerebrovascular disease and death from any cause.
(8) Includes ischaemic stroke, cerebral hemorrhage and other vascular events.
(9) Includes stroke, MI and vascular death.
(10) Includes stroke, MI and vascular death. Comparison group treated with aspirin OR Clopidogrel.

**Supplement Figure III** Effect of cilostazol on death from all causes by the end of follow-up

| Study or Subgroup | Cilostazol Events | Cilostazol Total | Control Events | Control Total | Weight | Peto Odds Ratio Peto, Fixed, 95% CI | Year |
|---|---|---|---|---|---|---|---|
| **7.4.1 Acute/subacute Cil v no Cil** | | | | | | | |
| **Subtotal (95% CI)** | | **0** | | **0** | | **Not estimable** | |
| Total events | 0 | | 0 | | | | |
| Heterogeneity: Not applicable | | | | | | | |
| Test for overall effect: Not applicable | | | | | | | |
| **7.4.2 Acute/subacute Cil+Asp or Clop v Asp or Clop** | | | | | | | |
| TOSS | 1 | 67 | 1 | 68 | 0.9% | 1.02 [0.06, 16.40] | 2005 |
| Nakamura | 0 | 38 | 0 | 38 | | Not estimable | 2012 |
| Shimizu | 2 | 251 | 1 | 256 | 1.3% | 1.99 [0.21, 19.24] | 2013 |
| ECLIPse | 0 | 100 | 0 | 103 | | Not estimable | 2013 |
| Ohnuki | 0 | 13 | 0 | 11 | | Not estimable | 2017 |
| **Subtotal (95% CI)** | | **469** | | **476** | **2.2%** | **1.52 [0.26, 8.83]** | |
| Total events | 3 | | 2 | | | | |
| Heterogeneity: Chi² = 0.14, df = 1 (P = 0.71); I² = 0% | | | | | | | |
| Test for overall effect: Z = 0.47 (P = 0.64) | | | | | | | |
| **7.4.3 Acute/subacute Cil v Asp or Clop** | | | | | | | |
| CAIST | 1 | 231 | 0 | 227 | 0.4% | 7.26 [0.14, 366.05] | 2011 |
| Lee | 0 | 40 | 0 | 40 | | Not estimable | 2017 |
| **Subtotal (95% CI)** | | **271** | | **267** | **0.4%** | **7.26 [0.14, 366.05]** | |
| Total events | 1 | | 0 | | | | |
| Heterogeneity: Not applicable | | | | | | | |
| Test for overall effect: Z = 0.99 (P = 0.32) | | | | | | | |
| **7.4.4 Acute/subacute Cil+Asp v Clop+Asp** | | | | | | | |
| TOSS-2 | 1 | 232 | 2 | 225 | 1.3% | 0.50 [0.05, 4.80] | 2011 |
| **Subtotal (95% CI)** | | **232** | | **225** | **1.3%** | **0.50 [0.05, 4.80]** | |
| Total events | 1 | | 2 | | | | |
| Heterogeneity: Not applicable | | | | | | | |
| Test for overall effect: Z = 0.61 (P = 0.54) | | | | | | | |
| **7.4.5 Chronic Cil v Pla** | | | | | | | |
| CSPS | 0 | 533 | 4 | 534 | 1.8% | 0.13 [0.02, 0.96] | 2006 |
| **Subtotal (95% CI)** | | **533** | | **534** | **1.8%** | **0.13 [0.02, 0.96]** | |
| Total events | 0 | | 4 | | | | |
| Heterogeneity: Not applicable | | | | | | | |
| Test for overall effect: Z = 2.00 (P = 0.05) | | | | | | | |
| **7.4.6 Chronic Cil+Asp or Clop v Asp or Clop** | | | | | | | |
| **Subtotal (95% CI)** | | **0** | | **0** | | **Not estimable** | |
| Total events | 0 | | 0 | | | | |
| Heterogeneity: Not applicable | | | | | | | |
| Test for overall effect: Not applicable | | | | | | | |
| **7.4.7 Chronic Cil v Asp or Clop** | | | | | | | |
| CASISP | 0 | 360 | 0 | 359 | | Not estimable | 2008 |
| Guo | 0 | 34 | 2 | 34 | 0.9% | 0.13 [0.01, 2.14] | 2009 |
| CSPS 2 | 13 | 1337 | 13 | 1335 | 11.5% | 1.00 [0.46, 2.16] | 2010 |
| Johkura | 0 | 57 | 0 | 49 | | Not estimable | 2012 |
| Sakurai | 0 | 11 | 0 | 9 | | Not estimable | 2013 |
| CATHARSIS | 0 | 79 | 0 | 724 | | Not estimable | 2015 |
| PICASSO | 28 | 766 | 31 | 768 | 25.3% | 0.90 [0.54, 1.52] | 2018 |
| CSPS.com | 47 | 932 | 90 | 947 | 56.6% | 0.52 [0.37, 0.73] | 2019 |
| LACI-1 | 0 | 42 | 0 | 15 | | Not estimable | 2019 |
| **Subtotal (95% CI)** | | **3618** | | **4240** | **94.2%** | **0.64 [0.49, 0.84]** | |
| Total events | 88 | | 136 | | | | |
| Heterogeneity: Chi² = 5.63, df = 3 (P = 0.13); I² = 47% | | | | | | | |
| Test for overall effect: Z = 3.22 (P = 0.001) | | | | | | | |
| **Total (95% CI)** | | **5123** | | **5742** | **100.0%** | **0.64 [0.49, 0.83]** | |
| Total events | 93 | | 144 | | | | |
| Heterogeneity: Chi² = 10.64, df = 8 (P = 0.22); I² = 25% | | | | | | | |
| Test for overall effect: Z = 3.33 (P = 0.0009) | | | | | | | |
| Test for subgroup differences: Chi² = 4.88, df = 4 (P = 0.30), I² = 18.0% | | | | | | | |

Peto Odds Ratio
Peto, Fixed, 95% CI

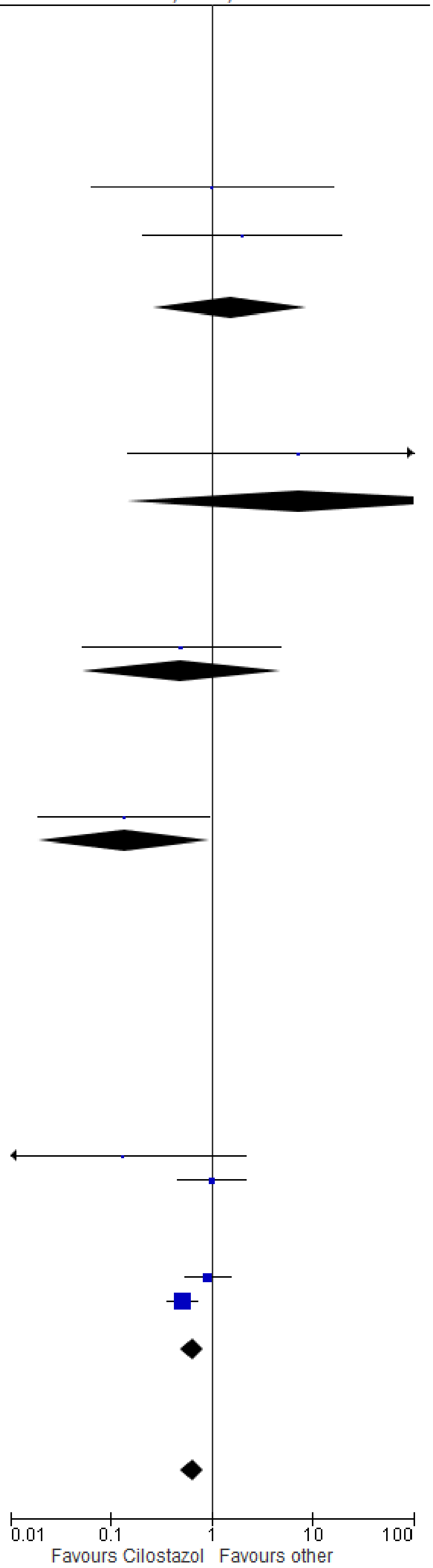

**Supplement Figure IV** Effect of cilostazol on cognition by the end of follow-up

| | Cilostazol | | | Control | | | | Std. Mean Difference | |
|---|---|---|---|---|---|---|---|---|---|
| **Study or Subgroup** | **Mean** | **SD** | **Total** | **Mean** | **SD** | **Total** | **Weight** | **IV, Random, 95% CI** | **Year** |
| **7.7.1 MMSE** | | | | | | | | | |
| Sakurai | 21.2 | 5.2 | 11 | 20.1 | 4.9 | 9 | 13.1% | 0.21 [-0.68, 1.09] | 2013 |
| CASID | 15.39 | 3.9 | 18 | 16.22 | 4.9 | 18 | 23.8% | -0.18 [-0.84, 0.47] | 2019 |
| **Subtotal (95% CI)** | | | **29** | | | **27** | **36.9%** | **-0.04 [-0.57, 0.48]** | |
| Heterogeneity: Tau² = 0.00; Chi² = 0.49, df = 1 (P = 0.49); I² = 0% | | | | | | | | | |
| Test for overall effect: Z = 0.17 (P = 0.87) | | | | | | | | | |
| **7.7.2 TMT-A** | | | | | | | | | |
| Sakurai | 78.6 | 39.5 | 11 | 85.1 | 22.5 | 9 | 13.1% | -0.19 [-1.07, 0.69] | 2013 |
| **Subtotal (95% CI)** | | | **11** | | | **9** | **13.1%** | **-0.19 [-1.07, 0.69]** | |
| Heterogeneity: Not applicable | | | | | | | | | |
| Test for overall effect: Z = 0.42 (P = 0.68) | | | | | | | | | |
| **7.7.3 ADAS-Cog** | | | | | | | | | |
| Sakurai | 20.3 | 10.5 | 11 | 20.1 | 8.3 | 9 | 13.2% | 0.02 [-0.86, 0.90] | 2013 |
| CASID | 25.78 | 8.1 | 18 | 24.78 | 7.4 | 18 | 23.9% | 0.13 [-0.53, 0.78] | 2019 |
| **Subtotal (95% CI)** | | | **29** | | | **27** | **37.0%** | **0.09 [-0.44, 0.61]** | |
| Heterogeneity: Tau² = 0.00; Chi² = 0.04, df = 1 (P = 0.85); I² = 0% | | | | | | | | | |
| Test for overall effect: Z = 0.33 (P = 0.74) | | | | | | | | | |
| **7.7.4 WSM-R (logical memory)** | | | | | | | | | |
| Sakurai | 5.6 | 4.7 | 11 | 4.2 | 3.5 | 9 | 13.0% | 0.32 [-0.57, 1.21] | 2013 |
| **Subtotal (95% CI)** | | | **11** | | | **9** | **13.0%** | **0.32 [-0.57, 1.21]** | |
| Heterogeneity: Not applicable | | | | | | | | | |
| Test for overall effect: Z = 0.70 (P = 0.48) | | | | | | | | | |
| **Total (95% CI)** | | | **80** | | | **72** | **100.0%** | **0.03 [-0.29, 0.35]** | |
| Heterogeneity: Tau² = 0.00; Chi² = 1.29, df = 5 (P = 0.94); I² = 0% | | | | | | | | | |
| Test for overall effect: Z = 0.20 (P = 0.84) | | | | | | | | | |
| Test for subgroup differences: Chi² = 0.77, df = 3 (P = 0.86), I² = 0% | | | | | | | | | |

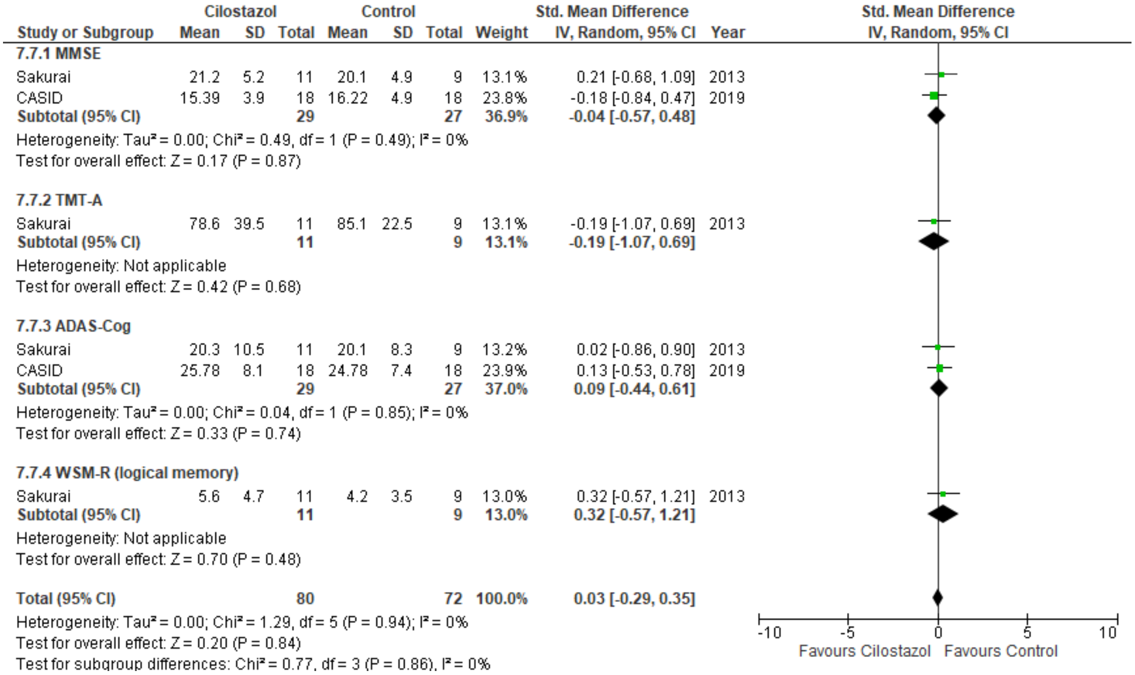

**Supplement Figure V**. Adverse symptoms including systemic bleeding

| Study or Subgroup | Cilostazol Events | Cilostazol Total | Control Events | Control Total | Weight | Peto Odds Ratio Peto, Fixed, 95% CI | Year |
|---|---|---|---|---|---|---|---|
| **7.6.1 Headache** | | | | | | | |
| CSPS | 68 | 533 | 17 | 534 | 8.4% | 3.69 [2.37, 5.74] | 2006 |
| CASISP | 49 | 360 | 19 | 359 | 6.6% | 2.64 [1.60, 4.35] | 2008 |
| Guo | 8 | 34 | 2 | 34 | 0.9% | 4.00 [1.06, 15.16] | 2009 |
| ARCC | 7 | 125 | 2 | 119 | 0.9% | 3.00 [0.79, 11.33] | 2010 |
| CSPS 2 | 313 | 1337 | 217 | 1335 | 45.8% | 1.57 [1.30, 1.90] | 2010 |
| CAIST | 114 | 231 | 71 | 227 | 11.9% | 2.11 [1.46, 3.07] | 2011 |
| Johkura | 0 | 57 | 0 | 49 | | Not estimable | 2012 |
| ECLIPse | 17 | 100 | 4 | 103 | 2.0% | 4.08 [1.66, 10.06] | 2013 |
| Shimizu | 4 | 251 | 1 | 256 | 0.5% | 3.42 [0.59, 19.88] | 2013 |
| Lee | 14 | 40 | 3 | 40 | 1.5% | 5.07 [1.75, 14.69] | 2017 |
| PICASSO | 114 | 766 | 72 | 768 | 17.6% | 1.68 [1.23, 2.28] | 2018 |
| CASID | 0 | 18 | 1 | 18 | 0.1% | 0.14 [0.00, 6.82] | 2019 |
| CSPS.com | 26 | 910 | 0 | 921 | 2.8% | 7.69 [3.55, 16.68] | 2019 |
| LACI-1 | 9 | 42 | 4 | 15 | 0.9% | 0.75 [0.19, 3.01] | 2019 |
| **Subtotal (95% CI)** | | **4804** | | **4778** | **100.0%** | **2.00 [1.76, 2.28]** | |
| Total events | 743 | | 413 | | | | |
| Heterogeneity: Chi² = 38.61, df = 12 (P = 0.0001); I² = 69% | | | | | | | |
| Test for overall effect: Z = 10.58 (P < 0.00001) | | | | | | | |
| **7.6.2 Dizziness** | | | | | | | |
| CSPS | 19 | 533 | 18 | 534 | 6.3% | 1.06 [0.55, 2.04] | 2006 |
| CASISP | 32 | 360 | 17 | 359 | 8.1% | 1.92 [1.08, 3.43] | 2008 |
| Guo | 2 | 34 | 0 | 34 | 0.3% | 7.62 [0.47, 124.34] | 2009 |
| CSPS 2 | 129 | 1337 | 97 | 1335 | 36.5% | 1.36 [1.04, 1.79] | 2010 |
| CAIST | 36 | 231 | 28 | 227 | 9.7% | 1.31 [0.77, 2.22] | 2011 |
| ECLIPse | 2 | 100 | 1 | 103 | 0.5% | 2.02 [0.21, 19.65] | 2013 |
| Lee | 4 | 40 | 2 | 40 | 1.0% | 2.04 [0.39, 10.65] | 2017 |
| PICASSO | 124 | 766 | 129 | 768 | 37.3% | 0.96 [0.73, 1.25] | 2018 |
| CASID | 1 | 18 | 0 | 18 | 0.2% | 7.39 [0.15, 372.38] | 2019 |
| **Subtotal (95% CI)** | | **3419** | | **3418** | **100.0%** | **1.22 [1.04, 1.44]** | |
| Total events | 349 | | 292 | | | | |
| Heterogeneity: Chi² = 9.36, df = 8 (P = 0.31); I² = 15% | | | | | | | |
| Test for overall effect: Z = 2.38 (P = 0.02) | | | | | | | |
| **7.6.3 Palpitations** | | | | | | | |
| CSPS | 28 | 533 | 2 | 534 | 7.7% | 5.95 [2.88, 12.30] | 2006 |
| CASISP | 5 | 360 | 35 | 359 | 10.0% | 0.20 [0.11, 0.39] | 2008 |
| CSPS 2 | 156 | 1337 | 71 | 1335 | 54.8% | 2.26 [1.72, 2.97] | 2010 |
| CAIST | 11 | 231 | 4 | 227 | 3.8% | 2.57 [0.92, 7.19] | 2011 |
| Nakamura | 3 | 38 | 0 | 38 | 0.8% | 7.81 [0.79, 77.38] | 2012 |
| Shimizu | 7 | 251 | 0 | 256 | 1.8% | 7.72 [1.74, 34.28] | 2013 |
| ECLIPse | 1 | 100 | 0 | 103 | 0.3% | 7.61 [0.15, 383.89] | 2013 |
| Lee | 9 | 40 | 3 | 40 | 2.7% | 3.20 [0.94, 10.82] | 2017 |
| PICASSO | 28 | 766 | 9 | 768 | 9.5% | 2.87 [1.50, 5.51] | 2018 |
| CSPS.com | 33 | 910 | 0 | 921 | 8.6% | 7.75 [3.89, 15.43] | 2019 |
| **Subtotal (95% CI)** | | **4566** | | **4581** | **100.0%** | **2.29 [1.87, 2.80]** | |
| Total events | 281 | | 124 | | | | |
| Heterogeneity: Chi² = 78.80, df = 9 (P < 0.00001); I² = 89% | | | | | | | |
| Test for overall effect: Z = 8.06 (P < 0.00001) | | | | | | | |
| **7.6.4 Tachycardia** | | | | | | | |
| CASISP | 22 | 360 | 7 | 359 | 16.4% | 2.93 [1.39, 6.15] | 2008 |
| Guo | 8 | 34 | 5 | 34 | 6.3% | 1.75 [0.53, 5.83] | 2009 |
| CSPS 2 | 89 | 1337 | 21 | 1335 | 62.2% | 3.62 [2.47, 5.31] | 2010 |
| Johkura | 0 | 57 | 0 | 49 | | Not estimable | 2012 |
| CSPS.com | 26 | 910 | 0 | 921 | 15.1% | 7.69 [3.55, 16.68] | 2019 |
| **Subtotal (95% CI)** | | **2698** | | **2698** | **100.0%** | **3.74 [2.77, 5.06]** | |
| Total events | 145 | | 33 | | | | |
| Heterogeneity: Chi² = 5.31, df = 3 (P = 0.15); I² = 43% | | | | | | | |
| Test for overall effect: Z = 8.60 (P < 0.00001) | | | | | | | |
| **7.6.5 Diarrhoea** | | | | | | | |
| CSPS 2 | 164 | 1337 | 85 | 1335 | 58.2% | 2.01 [1.55, 2.61] | 2010 |
| CAIST | 32 | 231 | 16 | 227 | 11.1% | 2.06 [1.13, 3.75] | 2011 |
| Lee | 1 | 40 | 1 | 40 | 0.5% | 1.00 [0.06, 16.27] | 2017 |
| PICASSO | 95 | 766 | 24 | 768 | 28.3% | 3.65 [2.51, 5.31] | 2018 |
| LACI-1 | 10 | 42 | 0 | 15 | 1.7% | 5.04 [1.08, 23.42] | 2019 |
| CASID | 1 | 18 | 0 | 18 | 0.3% | 7.39 [0.15, 372.38] | 2019 |
| **Subtotal (95% CI)** | | **2434** | | **2403** | **100.0%** | **2.42 [1.99, 2.96]** | |
| Total events | 303 | | 126 | | | | |
| Heterogeneity: Chi² = 8.46, df = 5 (P = 0.13); I² = 41% | | | | | | | |
| Test for overall effect: Z = 8.72 (P < 0.00001) | | | | | | | |
| **7.6.6 Constipation** | | | | | | | |
| CSPS 2 | 110 | 1337 | 155 | 1335 | 58.7% | 0.68 [0.53, 0.88] | 2010 |
| CAIST | 40 | 231 | 49 | 227 | 17.7% | 0.76 [0.48, 1.21] | 2011 |
| PICASSO | 39 | 766 | 64 | 768 | 23.6% | 0.60 [0.40, 0.89] | 2018 |
| **Subtotal (95% CI)** | | **2334** | | **2330** | **100.0%** | **0.68 [0.56, 0.82]** | |
| Total events | 189 | | 268 | | | | |
| Heterogeneity: Chi² = 0.65, df = 2 (P = 0.72); I² = 0% | | | | | | | |
| Test for overall effect: Z = 3.96 (P < 0.0001) | | | | | | | |
| **7.6.7 Nausea** | | | | | | | |
| CSPS | 14 | 533 | 7 | 534 | 17.6% | 1.98 [0.83, 4.69] | 2006 |
| CAIST | 39 | 231 | 30 | 227 | 50.0% | 1.33 [0.80, 2.22] | 2011 |
| PICASSO | 22 | 766 | 15 | 768 | 30.8% | 1.48 [0.77, 2.84] | 2018 |
| CASID (1) | 1 | 18 | 1 | 18 | 1.7% | 1.00 [0.06, 16.65] | 2019 |
| **Subtotal (95% CI)** | | **1548** | | **1547** | **100.0%** | **1.47 [1.02, 2.11]** | |
| Total events | 76 | | 53 | | | | |
| Heterogeneity: Chi² = 0.67, df = 3 (P = 0.88); I² = 0% | | | | | | | |
| Test for overall effect: Z = 2.07 (P = 0.04) | | | | | | | |
| **7.6.8 Bleeding** | | | | | | | |
| CASISP | 15 | 301 | 27 | 299 | 22.8% | 0.54 [0.29, 1.01] | 2008 |
| Guo | 0 | 34 | 5 | 34 | 2.7% | 0.12 [0.02, 0.73] | 2009 |
| CSPS 2 | 21 | 1337 | 8 | 1335 | 16.7% | 2.47 [1.19, 5.14] | 2010 |
| ARCC | 0 | 125 | 0 | 119 | | Not estimable | 2010 |
| TOSS-2 | 2 | 232 | 6 | 225 | 4.6% | 0.35 [0.09, 1.42] | 2011 |
| Nakamura | 0 | 38 | 1 | 38 | 0.6% | 0.14 [0.00, 6.82] | 2012 |
| Johkura | 0 | 57 | 0 | 49 | | Not estimable | 2012 |
| Shimizu | 0 | 251 | 0 | 256 | | Not estimable | 2013 |
| CATHARSIS | 3 | 83 | 1 | 80 | 2.3% | 2.67 [0.37, 19.31] | 2015 |
| Ohnuki | 0 | 13 | 0 | 11 | | Not estimable | 2017 |
| PICASSO | 27 | 766 | 49 | 768 | 42.2% | 0.55 [0.34, 0.87] | 2018 |
| LACI-1 | 4 | 42 | 3 | 15 | 2.8% | 0.38 [0.06, 2.28] | 2019 |
| CSPS.com (2) | 7 | 932 | 2 | 947 | 5.2% | 3.10 [0.84, 11.49] | 2019 |
| **Subtotal (95% CI)** | | **4211** | | **4176** | **100.0%** | **0.73 [0.54, 0.99]** | |
| Total events | 79 | | 102 | | | | |
| Heterogeneity: Chi² = 25.57, df = 8 (P = 0.001); I² = 69% | | | | | | | |
| Test for overall effect: Z = 2.02 (P = 0.04) | | | | | | | |

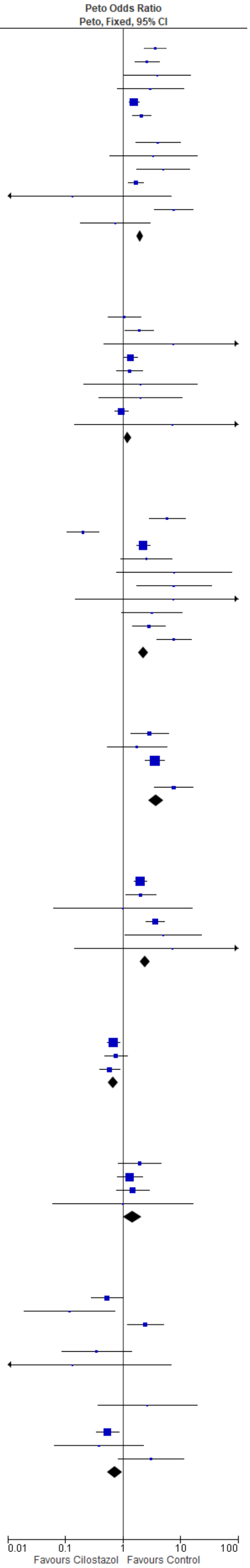


Test for subgroup differences: Chi² = 183.50, df = 7 (P < 0.00001), I² = 96.2%

Footnotes
(1) Includes vomitting.
(2) Minor bleeding.

## **Supplement Figure VI.** Sensitivity analyses

### A. Proportion with lacunar stroke

| | Cilostazol | | Control | | | Risk Ratio | |
|---|---|---|---|---|---|---|---|
| **Study or Subgroup** | **Events** | **Total** | **Events** | **Total** | **Weight** | **M-H, Random, 95% CI** | **Year** |
| **7.12.1 <39% lacunar or unknown** | | | | | | | |
| TOSS | 0 | 67 | 0 | 68 | | Not estimable | 2005 |
| CASISP | 11 | 360 | 15 | 359 | 5.0% | 0.73 [0.34, 1.57] | 2008 |
| Guo | 2 | 34 | 1 | 34 | 0.5% | 2.00 [0.19, 21.03] | 2009 |
| TOSS-2 | 10 | 232 | 6 | 225 | 2.9% | 1.62 [0.60, 4.37] | 2011 |
| Johkura | 0 | 57 | 0 | 49 | | Not estimable | 2012 |
| CATHARSIS | 4 | 83 | 6 | 80 | 1.9% | 0.64 [0.19, 2.19] | 2015 |
| Lee | 1 | 40 | 0 | 40 | 0.3% | 3.00 [0.13, 71.51] | 2017 |
| PICASSO | 40 | 766 | 73 | 768 | 21.0% | 0.55 [0.38, 0.80] | 2018 |
| **Subtotal (95% CI)** | | **1639** | | **1623** | **31.6%** | **0.72 [0.49, 1.07]** | |
| Total events | 68 | | 101 | | | | |
| Heterogeneity: Tau² = 0.04; Chi² = 5.84, df = 5 (P = 0.32); I² = 14% | | | | | | | |
| Test for overall effect: Z = 1.62 (P = 0.10) | | | | | | | |
| **7.12.2 >40% lacunar stroke** | | | | | | | |
| CSPS | 30 | 533 | 57 | 534 | 16.1% | 0.53 [0.34, 0.81] | 2006 |
| CSPS 2 | 72 | 1337 | 88 | 1335 | 31.9% | 0.82 [0.60, 1.11] | 2010 |
| CAIST | 4 | 231 | 5 | 227 | 1.7% | 0.79 [0.21, 2.89] | 2011 |
| Nakamura | 2 | 38 | 3 | 38 | 1.0% | 0.67 [0.12, 3.77] | 2012 |
| ECLIPse | 1 | 100 | 1 | 103 | 0.4% | 1.03 [0.07, 16.24] | 2013 |
| Shimizu | 3 | 251 | 4 | 256 | 1.3% | 0.76 [0.17, 3.38] | 2013 |
| Ohnuki | 0 | 13 | 0 | 11 | | Not estimable | 2017 |
| LACI-1 | 1 | 42 | 0 | 15 | 0.3% | 1.12 [0.05, 26.02] | 2019 |
| CSPS.com | 29 | 932 | 64 | 947 | 15.8% | 0.46 [0.30, 0.71] | 2019 |
| **Subtotal (95% CI)** | | **3477** | | **3466** | **68.4%** | **0.64 [0.52, 0.79]** | |
| Total events | 142 | | 222 | | | | |
| Heterogeneity: Tau² = 0.00; Chi² = 5.95, df = 7 (P = 0.55); I² = 0% | | | | | | | |
| Test for overall effect: Z = 4.18 (P < 0.0001) | | | | | | | |
| **Total (95% CI)** | | **5116** | | **5089** | **100.0%** | **0.65 [0.55, 0.77]** | |
| Total events | 210 | | 323 | | | | |
| Heterogeneity: Tau² = 0.00; Chi² = 11.82, df = 13 (P = 0.54); I² = 0% | | | | | | | |
| Test for overall effect: Z = 4.94 (P < 0.00001) | | | | | | | |
| Test for subgroup differences: Chi² = 0.27, df = 1 (P = 0.60), I² = 0% | | | | | | | |

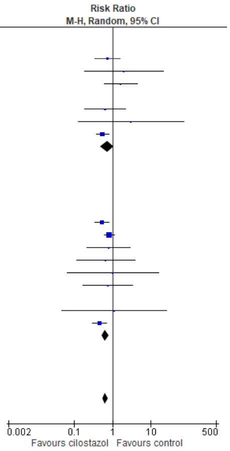


### B Ischaemic stroke by time to treatment

| | Cilostazol | | Control | | | Peto Odds Ratio | |
|---|---|---|---|---|---|---|---|
| **Study or Subgroup** | **Events** | **Total** | **Events** | **Total** | **Weight** | **Peto, Fixed, 95% CI** | **Year** |
| **7.9.1 Acute/subacute any Cil v any no Cil** | | | | | | | |
| TOSS | 0 | 67 | 0 | 68 | | Not estimable | 2005 |
| TOSS-2 | 10 | 232 | 6 | 225 | 3.2% | 1.62 [0.60, 4.40] | 2011 |
| CAIST | 4 | 231 | 5 | 227 | 1.8% | 0.78 [0.21, 2.93] | 2011 |
| Nakamura | 2 | 38 | 3 | 38 | 1.0% | 0.66 [0.11, 3.97] | 2012 |
| Shimizu (1) | 3 | 251 | 4 | 256 | 1.4% | 0.76 [0.17, 3.39] | 2013 |
| ECLIPse | 1 | 100 | 1 | 103 | 0.4% | 1.03 [0.06, 16.59] | 2013 |
| Lee (2) | 1 | 40 | 0 | 40 | 0.2% | 7.39 [0.15, 372.38] | 2017 |
| Ohnuki | 0 | 13 | 0 | 11 | | Not estimable | 2017 |
| **Subtotal (95% CI)** | | **972** | | **968** | **8.0%** | **1.10 [0.58, 2.05]** | |
| Total events | 21 | | 19 | | | | |
| Heterogeneity: Chi² = 2.30, df = 5 (P = 0.81); I² = 0% | | | | | | | |
| Test for overall effect: Z = 0.28 (P = 0.78) | | | | | | | |
| **7.9.5 Chronic any Cil v any no Cil** | | | | | | | |
| CSPS | 30 | 533 | 57 | 534 | 16.5% | 0.51 [0.33, 0.79] | 2006 |
| CASISP | 11 | 360 | 15 | 359 | 5.2% | 0.72 [0.33, 1.59] | 2008 |
| Guo | 2 | 34 | 1 | 34 | 0.6% | 1.99 [0.20, 19.78] | 2009 |
| CSPS 2 | 72 | 1337 | 88 | 1335 | 31.0% | 0.81 [0.59, 1.11] | 2010 |
| Johkura | 0 | 57 | 0 | 49 | | Not estimable | 2012 |
| Sakurai (3) | 0 | 11 | 0 | 9 | | Not estimable | 2013 |
| CATHARSIS | 4 | 83 | 6 | 80 | 1.9% | 0.63 [0.18, 2.25] | 2015 |
| PICASSO | 40 | 766 | 55 | 768 | 18.4% | 0.72 [0.47, 1.08] | 2018 |
| LACI-1 (4) | 1 | 42 | 0 | 15 | 0.2% | 3.89 [0.05, 332.98] | 2019 |
| CSPS.com (5) | 29 | 932 | 64 | 947 | 18.2% | 0.46 [0.30, 0.70] | 2019 |
| **Subtotal (95% CI)** | | **4155** | | **4130** | **92.0%** | **0.65 [0.54, 0.78]** | |
| Total events | 189 | | 286 | | | | |
| Heterogeneity: Chi² = 7.37, df = 7 (P = 0.39); I² = 5% | | | | | | | |
| Test for overall effect: Z = 4.59 (P < 0.00001) | | | | | | | |
| **Total (95% CI)** | | **5127** | | **5098** | **100.0%** | **0.68 [0.57, 0.81]** | |
| Total events | 210 | | 305 | | | | |
| Heterogeneity: Chi² = 12.14, df = 13 (P = 0.52); I² = 0% | | | | | | | |
| Test for overall effect: Z = 4.32 (P < 0.0001) | | | | | | | |
| Test for subgroup differences: Chi² = 2.47, df = 1 (P = 0.12), I² = 59.5% | | | | | | | |

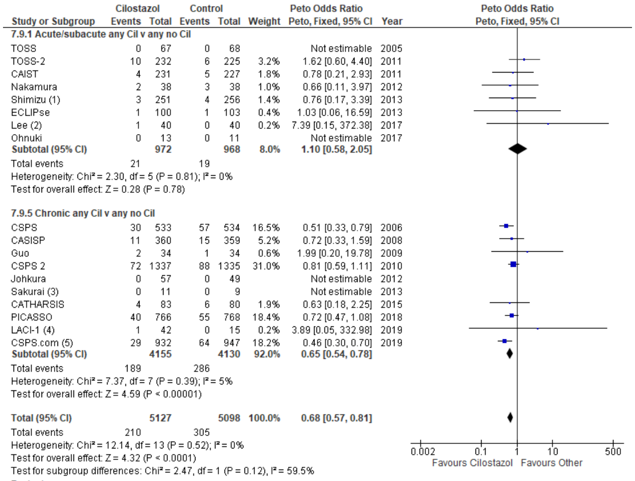


Footnotes
(1) Comparison group received placebo OR no treatment.
(2) Comparison group received aspirin OR placebo.
(3) Comparison group received aspirin AND Clopidogrel.
(4) Comparison group received aspirin OR Clopidogrel.
(5) Comparison group treated with aspirin OR Clopidogrel.

### C Ischaemic stroke by additional antiplatelets

| | Cilostazol | | Control | | | Peto Odds Ratio | |
|---|---|---|---|---|---|---|---|
| **Study or Subgroup** | **Events** | **Total** | **Events** | **Total** | **Weight** | **Peto, Fixed, 95% CI** | **Year** |
| **7.10.1 Cil v no Cil, no antiplatelet** | | | | | | | |
| CSPS | 30 | 533 | 57 | 534 | 16.5% | 0.51 [0.33, 0.79] | 2006 |
| **Subtotal (95% CI)** | | **533** | | **534** | **16.5%** | **0.51 [0.33, 0.79]** | |
| Total events | 30 | | 57 | | | | |
| Heterogeneity: Not applicable | | | | | | | |
| Test for overall effect: Z = 3.01 (P = 0.003) | | | | | | | |
| **7.10.2 Cil+Asp or Clop v Asp or Clop** | | | | | | | |
| TOSS | 0 | 67 | 0 | 68 | | Not estimable | 2005 |
| Nakamura | 2 | 38 | 3 | 38 | 1.0% | 0.66 [0.11, 3.97] | 2012 |
| ECLIPse | 1 | 100 | 1 | 103 | 0.4% | 1.03 [0.06, 16.59] | 2013 |
| Shimizu (1) | 3 | 251 | 4 | 256 | 1.4% | 0.76 [0.17, 3.39] | 2013 |
| CATHARSIS | 4 | 83 | 6 | 80 | 1.9% | 0.63 [0.18, 2.25] | 2015 |
| Ohnuki | 0 | 13 | 0 | 11 | | Not estimable | 2017 |
| CSPS.com (2) | 29 | 932 | 64 | 947 | 18.2% | 0.46 [0.30, 0.70] | 2019 |
| LACI-1 (3) | 1 | 42 | 0 | 15 | 0.2% | 3.89 [0.05, 332.98] | 2019 |
| **Subtotal (95% CI)** | | **1526** | | **1518** | **23.1%** | **0.51 [0.35, 0.74]** | |
| Total events | 40 | | 78 | | | | |
| Heterogeneity: Chi² = 1.73, df = 5 (P = 0.88); I² = 0% | | | | | | | |
| Test for overall effect: Z = 3.57 (P = 0.0004) | | | | | | | |
| **7.10.3 Cil v Asp or Clop** | | | | | | | |
| CASISP | 11 | 360 | 15 | 359 | 5.2% | 0.72 [0.33, 1.59] | 2008 |
| Guo | 2 | 34 | 1 | 34 | 0.6% | 1.99 [0.20, 19.78] | 2009 |
| CSPS 2 | 72 | 1337 | 88 | 1335 | 31.0% | 0.81 [0.59, 1.11] | 2010 |
| TOSS-2 | 10 | 232 | 6 | 225 | 3.2% | 1.62 [0.60, 4.40] | 2011 |
| CAIST | 4 | 231 | 5 | 227 | 1.8% | 0.78 [0.21, 2.93] | 2011 |
| Johkura | 0 | 57 | 0 | 49 | | Not estimable | 2012 |
| Sakurai (4) | 0 | 11 | 0 | 9 | | Not estimable | 2013 |
| Lee (5) | 1 | 40 | 0 | 40 | 0.2% | 7.39 [0.15, 372.38] | 2017 |
| PICASSO | 40 | 766 | 55 | 768 | 18.4% | 0.72 [0.47, 1.08] | 2018 |
| **Subtotal (95% CI)** | | **3068** | | **3046** | **60.4%** | **0.81 [0.65, 1.02]** | |
| Total events | 140 | | 170 | | | | |
| Heterogeneity: Chi² = 4.10, df = 6 (P = 0.66); I² = 0% | | | | | | | |
| Test for overall effect: Z = 1.77 (P = 0.08) | | | | | | | |
| **Total (95% CI)** | | **5127** | | **5098** | **100.0%** | **0.68 [0.57, 0.81]** | |
| Total events | 210 | | 305 | | | | |
| Heterogeneity: Chi² = 12.14, df = 13 (P = 0.52); I² = 0% | | | | | | | |
| Test for overall effect: Z = 4.32 (P < 0.0001) | | | | | | | |
| Test for subgroup differences: Chi² = 6.31, df = 2 (P = 0.04), I² = 68.3% | | | | | | | |

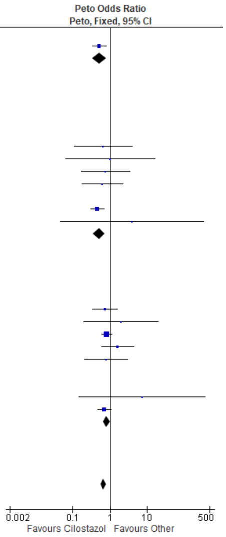


Footnotes
(1) Comparison group received placebo OR no treatment.
(2) Comparison group treated with aspirin OR Clopidogrel.
(3) Comparison group received aspirin OR Clopidogrel.
(4) Comparison group received aspirin AND Clopidogrel.
(5) Comparison group received aspirin OR placebo.

**Supplement Figure VII**. Chart of proportions of papers meeting CONSORT quality criteria

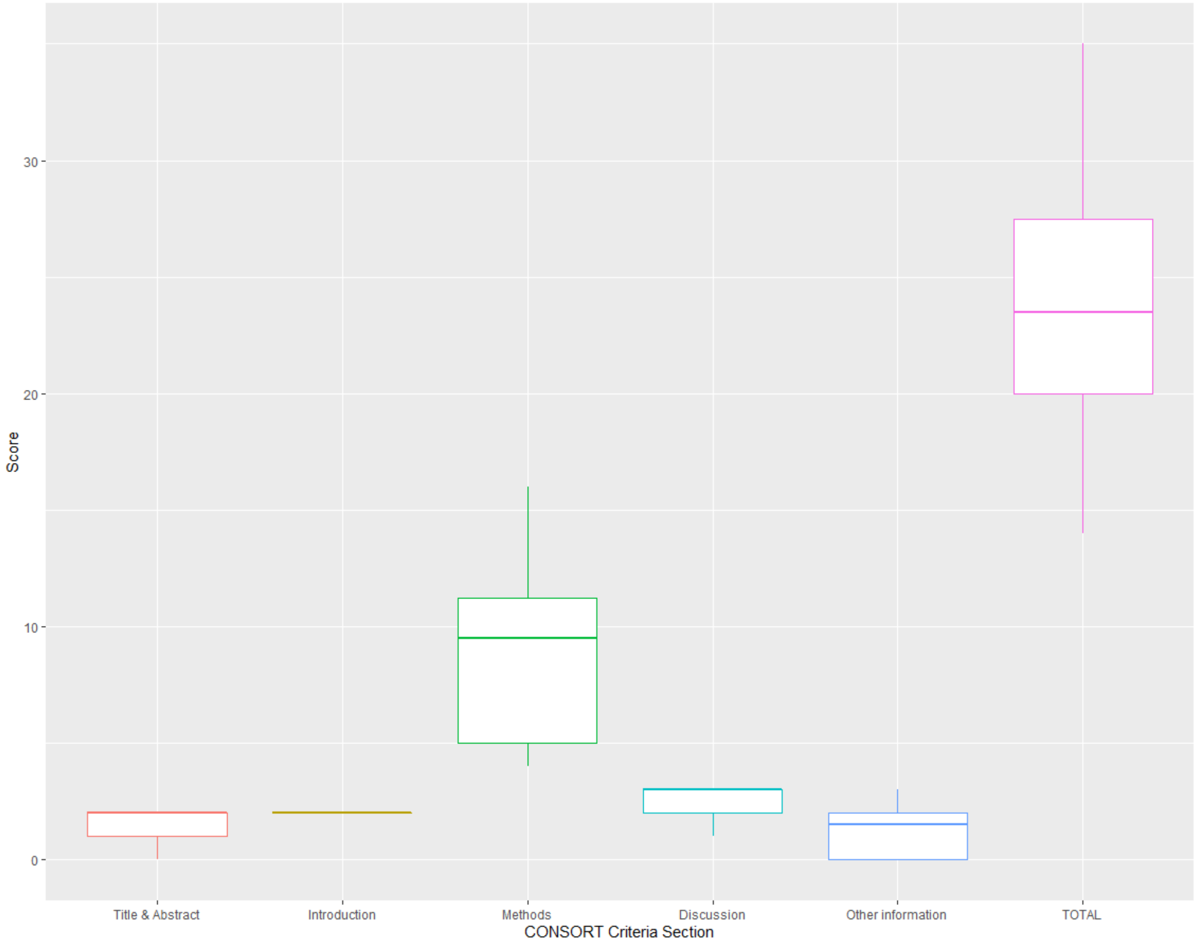

**Supplement Figure VIII**. Funnel plots

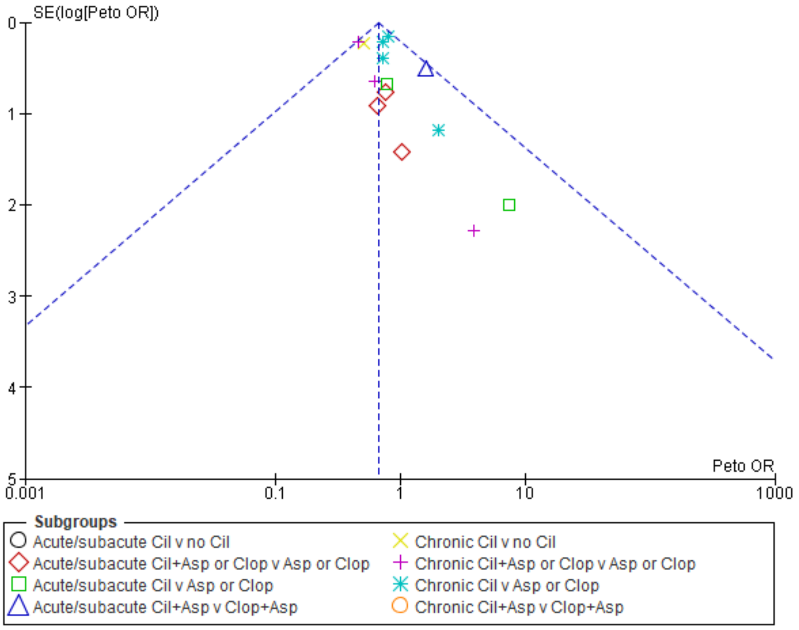

**References to Supplement**


1. Lee JH, Cha JK, Lee SJ, Ha SW, Kwon SU. Addition of cilostazol reduces biological aspirin resistance in aspirin users with ischaemic stroke: A double-blind randomized clinical trial. *European Journal of Neurology*. 2010;17:434-442
2. Lee YS, Bae HJ, Kang DW, Lee SH, Yu K, Park JM, et al. Cilostazol in acute ischemic stroke treatment (caist trial): A randomized double-blind non-inferiority trial. *Cerebrovasc Dis*. 2011;32:65-71
3. Lee JY, Lee H, Yoo H, Choi JS, Jung HY, Yoon E, et al. Efficacy of cilostazol administration in alzheimer's disease patients with white matter lesions: A positron-emission tomography study. *Neurotherapeutics*. 2019;16:394-403
4. Huang Y, Cheng Y, Wu J, Li Y, Xu E, Hong Z, et al. Cilostazol as an alternative to aspirin after ischaemic stroke: A randomised, double-blind, pilot study. *Lancet Neurol*. 2008;7:494-499
5. Uchiyama S, Sakai N, Toi S, Ezura M, Okada Y, Takagi M, et al. Final results of cilostazol-aspirin therapy against recurrent stroke with intracranial artery stenosis (catharsis). *Cerebrovasc Dis Extra*. 2015;5:1-13
6. Gotoh F, Tohgi H, Hirai S, Terashi A, Fukuuchi Y, Otomo E, et al. Cilostazol stroke prevention study: A placebo-controlled double-blind trial for secondary prevention of cerebral infarction. *J Stroke Cerebrovasc Dis*. 2000;9:147-157
7. Shinohara Y, Gotoh F, Tohgi H, Hirai S, Terashi A, Fukuuchi Y, et al. Antiplatelet cilostazol is beneficial in diabetic and/or hypertensive ischemic stroke patients. Subgroup analysis of the cilostazol stroke prevention study. *Cerebrovasc Dis*. 2008;26:63-70
8. Shinohara Y. Antiplatelet cilostazol is effective in the prevention of pneumonia in ischemic stroke patients in the chronic stage. *Cerebrovascular Diseases*. 2006;22:57-60
9. Shinohara Y, Katayama Y, Uchiyama S, Yamaguchi T, Handa S, Matsuoka K, et al. Cilostazol for prevention of secondary stroke (csps 2): An aspirin-controlled, double-blind, randomised non-inferiority trial. *Lancet Neurol*. 2010;9:959-968
10. Uchiyama S. Results of the cilostazol stroke prevention study ii (csps ii): A randomized controlled trial for the comparison of cilostazol and aspirin in stroke patients. *Clinical Neurology*. 2010;50:832-834
11. Uchiyama S, Shinohara Y, Katayama Y, Yamaguchi T, Handa S, Matsuoka K, et al. Benefit of cilostazol in patients with high risk of bleeding: Subanalysis of cilostazol stroke prevention study 2. *Cerebrovascular Diseases*. 2014;37:296-303
12. Toyoda K, Uchiyama S, Yamaguchi T, Easton JD, Kimura K, Hoshino H, et al. Dual antiplatelet therapy using cilostazol for secondary prevention in patients with high-risk ischaemic stroke in japan: A multicentre, open-label, randomised controlled trial. *Lancet Neurol*. 2019;18:539-548
13. Toyoda K, Uchiyama S, Hoshino H, Kimura K, Origasa H, Naritomi H, et al. Protocol for cilostazol stroke prevention study for antiplatelet combination (csps.Com): A randomized, open-label, parallel-group trial. *Int J Stroke*. 2015;10:253-258
14. Han SW, Lee SS, Kim SH, Lee JH, Kim GS, Kim OJ, et al. Effect of cilostazol in acute lacunar infarction based on pulsatility index of transcranial doppler (eclipse): A multicenter, randomized, double-blind, placebo-controlled trial. *Eur Neurol*. 2013;69:33-40
15. Han SW, Song TJ, Bushnell CD, Lee SS, Kim SH, Lee JH, et al. Cilostazol decreases cerebral arterial pulsatility in patients with mild white matter hyperintensities: Subgroup analysis from the effect of cilostazol in acute lacunar infarction based on pulsatility index of transcranial doppler (eclipse) study. *Cerebrovasc Dis*. 2014;38:197-203
16. Guo JJ, Xu E, Lin QY, Zeng GL, Xie HF. Effect of cilostazol on cerebral arteries in secondary prevention of ischemic stroke. *Neurosci Bull*. 2009;25:383-390
17. Johkura K, Yoshida TN, Kudo Y, Nakae Y, Momoo T, Kuroiwa Y. Cilostazol versus aspirin therapy in patients with chronic dizziness after ischemic stroke. *Clinical Neurology and Neurosurgery*. 2012;114:876-880
18. Blair GW, Appleton JP, Flaherty K, Doubal F, Sprigg N, Dooley R, et al. Tolerability, safety and intermediary pharmacological effects of cilostazol and isosorbide mononitrate, alone and combined, in patients with lacunar ischaemic stroke: The lacunar intervention-1 (laci-1) trial, a randomised clinical trial. *EClinicalMedicine* 2019;11:34-43
19. Blair GW, Appleton JP, Law ZK, Doubal F, Flaherty K, Dooley R, et al. Preventing cognitive decline and dementia from cerebral small vessel disease: The laci-1 trial. Protocol and statistical analysis plan of a phase iia dose escalation trial testing tolerability, safety and effect on intermediary endpoints of isosorbide mononitrate and cilostazol, separately and in combination. *Int J Stroke*. 2018;13:530-538
20. Appleton JP, Blair GW, Flaherty K, Law ZK, May J, Woodhouse LJ, et al. Effects of isosorbide mononitrate and/or cilostazol on haematological markers, platelet function and haemodynamics in

patients with lacunar ischaemic stroke: Safety data from the lacunar intervention-1 (laci-1) trial. *Front Neurol*. 2019;10
21. Lee SJ, Lee JS, Choi MH, Lee SE, Shin DH, Hong JM. Cilostazol improves endothelial function in acute cerebral ischemia patients: A double-blind placebo controlled trial with flow-mediated dilation technique. *Bmc Neurology*. 2017;17
22. Nakamura T, Tsuruta S, Uchiyama S. Cilostazol combined with aspirin prevents early neurological deterioration in patients with acute ischemic stroke: A pilot study. *J Neurol Sci*. 2012;313:22-26
23. Ohnuki Y, Kohara S, Shimizu M, Takizawa S. Dual therapy with aspirin and cilostazol may improve platelet aggregation in noncardioembolic stroke patients: A pilot study. *Internal Medicine*. 2017;56:1307-1313
24. Hong KS, Kim BJ, Lee JY, Kwon SU. Rationale and design of the prevention of cardiovascular events in ischemic stroke patients with high risk of cerebral hemorrhage (picasso) study: A randomized controlled trial. *Int J Stroke*. 2015;10:1153-1158
25. Kim BJ, Lee EJ, Kwon SU, Park JH, Kim YJ, Hong KS, et al. Prevention of cardiovascular events in asian patients with ischaemic stroke at high risk of cerebral haemorrhage (picasso): A multicentre, randomised controlled trial. *Lancet Neurol*. 2018;17:509-518
26. Kwon HS, Cho AH, Lee MH, Lee D, Jeong D, Choi C, et al. Evolution of acute lacunar lesions in terms of size and shape: A picasso sub-study. *J Neurol*. 2019;266:766-772
27. Sakurai H, Hanyu H, Sato T, Kume K, Hirao K, Kanetaka H, et al. Effects of cilostazol on cognition and regional cerebral blood flow in patients with alzheimer's disease and cerebrovascular disease: A pilot study. *Geriatrics & Gerontology International*. 2013;13:90-97
28. Shimizu H, Tominaga T, Ogawa A, Kayama T, Mizoi K, Saito K, et al. Cilostazol for the prevention of acute progressing stroke: A multicenter, randomized controlled trial. *J Stroke Cerebrovasc Dis*. 2013;22:449-456
29. Kwon SU, Cho YJ, Koo JS, Bae HJ, Lee YS, Hong KS, et al. Cilostazol prevents the progression of the symptomatic intracranial arterial stenosis: The multicenter double-blind placebo-controlled trial of cilostazol in symptomatic intracranial arterial stenosis. *Stroke*. 2005;36:782-786
30. Kwon SU, Hong KS, Kang DW, Park JM, Lee JH, Cho YJ, et al. Efficacy and safety of combination antiplatelet therapies in patients with symptomatic intracranial atherosclerotic stenosis. *Stroke*. 2011;42:2883-2890
